# Quantification of blood flow index in diffuse correlation spectroscopy using a robust deep learning method

**DOI:** 10.1117/1.JBO.29.1.015004

**Published:** 2024-01-27

**Authors:** Quan Wang, Mingliang Pan, Zhenya Zang, David Day-Uei Li

**Affiliations:** University of Strathclyde, Department of Biomedical Engineering, Faculty of Engineering, Glasgow, United Kingdom

**Keywords:** deep learning, blood flow, diffuse correlation spectroscopy

## Abstract

**Significance:**

Diffuse correlation spectroscopy (DCS) is a powerful, noninvasive optical technique for measuring blood flow. Traditionally the blood flow index (BFi) is derived through nonlinear least-square fitting the measured intensity autocorrelation function (ACF). However, the fitting process is computationally intensive, susceptible to measurement noise, and easily influenced by optical properties (absorption coefficient $\mu_{a}$ and reduced scattering coefficient $\mu_{s}^{\prime }$) and scalp and skull thicknesses.

**Aim:**

We aim to develop a data-driven method that enables rapid and robust analysis of multiple-scattered light’s temporal ACFs. Moreover, the proposed method can be applied to a range of source–detector distances instead of being limited to a specific source–detector distance.

**Approach:**

We present a deep learning architecture with one-dimensional convolution neural networks, called DCS neural network (DCS-NET), for BFi and coherent factor (β) estimation. This DCS-NET was performed using simulated DCS data based on a three-layer brain model. We quantified the impact from physiologically relevant optical property variations, layer thicknesses, realistic noise levels, and multiple source–detector distances (5, 10, 15, 20, 25, and 30 mm) on BFi and β estimations among DCS-NET, semi-infinite, and three-layer fitting models.

**Results:**

DCS-NET shows a much faster analysis speed, around 17,000-fold and 32-fold faster than the traditional three-layer and semi-infinite models, respectively. It offers higher intrinsic sensitivity to deep tissues compared with fitting methods. DCS-NET shows excellent anti-noise features and is less sensitive to variations of $\mu_{a}$ and $\mu_{s}^{\prime }$ at a source–detector separation of 30 mm. Also, we have demonstrated that relative BFi (rBFi) can be extracted by DCS-NET with a much lower error of 8.35%. By contrast, the semi-infinite and three-layer fitting models result in significant errors in rBFi of 43.76% and 19.66%, respectively.

**Conclusions:**

DCS-NET can robustly quantify blood flow measurements at considerable source–detector distances, corresponding to much deeper biological tissues. It has excellent potential for hardware implementation, promising continuous real-time blood flow measurements.

## 1 Introduction

Cerebral blood flow (CBF) is essential for monitoring metabolic oxygenation,^1^^,^^2^ neurovascular coupling,^3^^,^^4^ and metabolic response to functional stimuli.^5^^,^^6^ For example, CBF abnormalities are caused by ischemic strokes,^7^ head trauma,^8^ or brain injury.^9^^,^^10^ There are several blood flow measurement techniques, including computed tomography,^11^ magnetic resonance imaging,^12^ and positron emission tomography.^13^ However, although they are well-established, they cannot provide continuous, long-term measurements at the bedside. Laser Doppler flowmetry can measure microvascular blood flow but only probe shallow tissues.^14^ Doppler ultrasound techniques can only measure blood flow in larger vasculatures and are unsuitable for longitudinal monitoring for unstable probe orientations.^15^ Near-infrared diffuse optical methods are becoming popular in blood flow measurements as they are noninvasive, nonionized, portable, and faster. Among them is diffuse correlation spectroscopy (DCS),^16^^,^^17^ using a laser with a long coherence length (>5  m) to illuminate tissue surfaces and collect remitted scattered light at a distance, typically 1 to 3 cm, away from the incident position. The scattered light from flowing red blood cells causes a speckle pattern fluctuating at a rate proportional to the flow rate. This blood-flow-dependent information can be quantified based on the normalized temporal intensity autocorrelation function (ACF) $g_{2}(\tau )\equiv \frac{\left\langle I(t)I(t+\tau )\right\rangle }{{\left\langle I(t)\right\rangle }^{2}}$, where I(t) is the measured scattered light and τ is the correlation lag time.^17^^,^^18^ DCS can measure blood flow *in vivo* in small animals,^19^^,^^20^ human brains,^16^ and muscles.^21^ Traditionally, to derive blood flow index (BFi), the measured $g_{2}(\tau )$ is fitted with a homogenous semi-infinite one-layer analytical model^22^ or the Monte Carlo model.^23^ This fitting process typically utilizes nonlinear least-square methods (NLSMs) with Levenberg–Marquardt optimization or trust-region-reflective methods.^24^^–^^26^ However, treating biological tissues with a homogenous semi-infinite model is not quite realistic, as significant signal contamination from superficial tissue layers (e.g., scalp/skull) occurs when measuring deep flow in the brain. Research has been conducted to minimize the discrepancy, with the diffusion equation for layered geometries developed for fitting methods, including two-^27^^,^^28^ and three-layer analytical models.^29^^,^^30^ Unfortunately, multilayer models highly rely on a priori knowledge of each layer’s optical properties (namely the absorption coefficient $\mu_{a}$ and reduced scattering coefficient $\mu_{s}^{\prime }$) and thickness to estimate blood flow within each layer. Commonly, layer optical properties and thicknesses are assumed from literature, and the errors in these assumed values can lead to significant errors in brain blood flow estimations. Additionally, the multilayer model is susceptible to measurement noise, especially for the three-layer model, although its accuracy in BFi estimations has been validated.^24^^,^^31^ Moreover, these methods are iterative and time-consuming. To overcome these limitations, the N’th-order linear (NL) algorithm,^32^^,^^33^ least-absolute minimization (L1 norm), and the support vector regression (SVR)^34^ were proposed. However, under the NL framework, BFi extraction is significantly influenced by the linear regression approach adopted.^34^ Although L1 norm and SVR are new approaches to processing DCS data, they are sensitive to signal deviations.^35^^,^^36^ Additionally, the BFi computing time is 28.07 and 52.93 s (using L1 norm and SVR, respectively), still slow for practical applications, particularly for real-time monitoring.^34^

Deep learning, an increasingly popular method, has been widely applied to biomedical time sequence data, including electroencephalogram (EEG) and electrocardiogram (ECG),^37^^,^^38^ but has yet to be broadly used in DCS. Very recently, Zhang et al.^39^ proposed the first recurrent neural network (RNN) regression model to DCS, followed by 2D convolution neural networks (2D CNNs),^40^ long short-term memory (LSTM),^41^ and ConvGRU.^42^ LSTM, as a typical RNN structure, has proven stable and robust for quantifying relative blood flow in previous studies in phantom and *in vivo* experiments.^41^ 2D CNN, on the other hand, tends to require large training datasets for complex structures, demanding massive memory resources. ConvGRU, the newest deep learning method introduced to DCS, has also exhibited excellent performances in BFi extraction.

Nevertheless, all existing algorithms are designed for a single source–detector distance (ρ), corresponding to a specific depth in biological tissues. To accommodate a wider range of ρ, retraining the model becomes necessary. Inspired by a recently published one-dimensional convolutional neural network (1D CNN)^43^ for fluorescence lifetime imaging (FLIM), we proposed the DCS neural network (DCS-NET) based on 1D CNN for quantifying the coherent factor β and BFi.

**Fig. 1:**
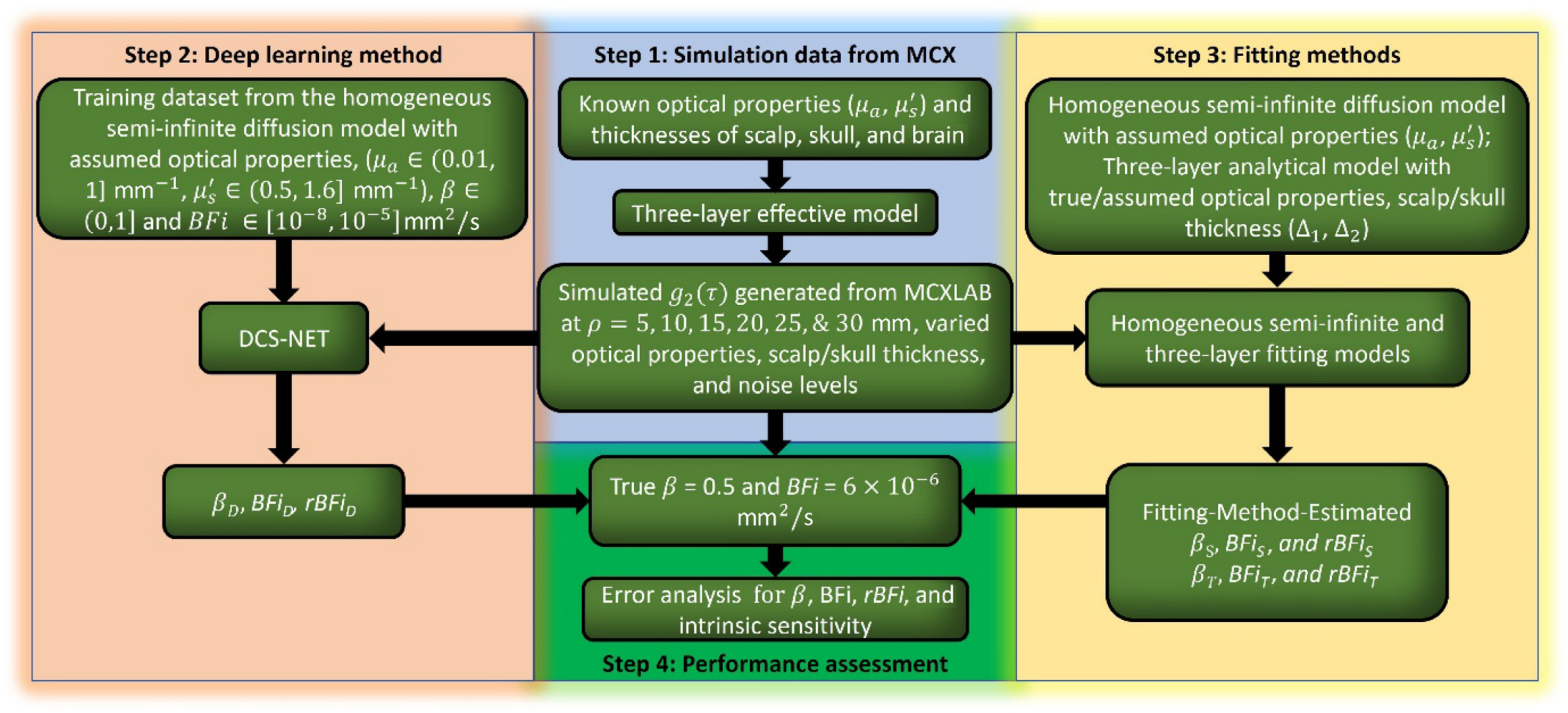
Flowchart of the proposed analysis. Step 1 generates the ACF $g_{2}(\tau )$ from MCX at different source–detector distances (5, 10, 15, 20, 25, and 30 mm), optical properties ($\mu_{a1,2,3}$, $\mu_{s1,2,3}^{\prime }$), scalp/skull thicknesses ($\Delta_{1}$, $\Delta_{2}$), and different noise levels using the three-layer slab. Step 2 obtains training datasets containing noise. The datasets are generated using a semi-infinite diffusion model with $\mu_{a}\in (0.01,1]{\;\mathrm{mm}}^{-1}$, $\mu_{s}^{\prime }\in (0.5,\;1.6]\;{\mathrm{mm}}^{-1}$, β∈(0,1], and $\mathrm{BFi}\in [10^{-8},10^{-5}]{\;\mathrm{mm}}^{2}/s$. Then, the simulated $g_{2}(\tau )$ from step 1 is analyzed by the pretrained model to predict β and BFi. Step 3 fits the simulated data from step 1 with semi-infinite and three-layer models with known/assumed optical properties/thicknesses to extract β and BFi. Step 4 assesses BFi and β estimations and concludes the intrinsic sensitivity and errors in terms of the variations in $\mu_{a}$, $\mu_{s}^{\prime }$, $\Delta_{1}$, and $\Delta_{2}$.

The primary objective of this work is to present and evaluate an artificial intelligence (AI) framework, called DCS-NET, in β and BFi estimations. We established the Monte Carlo simulation model based on the open-source tool Monte Carlo eXtreme (MCX) developed by Fang and Boas^44^ to generate $g_{2}(\tau )$ emulating experiment data. The DCS-NET training, validation, and testing datasets are from the semi-infinite geometry model.^22^ We investigated DCS-NET’s performance on absolute BFi and relative BFI (rBFi)’s estimations and compared them with semi-infinite and three-layer model fitting methods. To best link our work with actual outcomes expected in practice, we modeled DCS measurement noise based on realistic experimental conditions, considering various noise levels controlled by the integration time ($T_{\mathrm{int}}$). We define a metric that accounts for the intrinsic sensitivity of the brain blood flow and evaluate it between DCS-NET and traditional fitting methods. We also show BFi estimation errors induced by the inaccurate assumptions about layer optical properties and thicknesses when using fitting methods based on the semi-infinite and three-layer solutions of the correlation diffusion equation. Figure 1 summarizes the main concept of our work. All essential parameters are defined in Table 6 in the Appendix to facilitate our discussion.

## 2 Methods

### 2.1 DCS Theory

The transport of the unnormalized electric field auto-correlation function, $G_{1}(\rho ,\tau )\equiv \langle E^{*}(\rho ,t)\cdot E(\rho ,t)\rangle$, is well described by the correlation diffusion equation:^17^^,^^45^

$$(-\frac{1}{3\mu_{s}^{\prime }}\nabla^{2}+\mu_{a}+\frac{1}{3}\alpha k_{0}^{2}\mu_{s}^{\prime }\langle \Delta r^{2}(\tau )\rangle )G_{1}(\rho ,\tau )=S(\rho ),$$ (1)

where $k_{0}=2\pi n/\lambda$ is the wavenumber of light, n and λ are the refractive index and wavelength in the scattering medium, respectively. α is the fraction of dynamic photon scattering events in the medium. $\left\langle \Delta r^{2}(\tau )\right\rangle$ is the mean squared displacement of scatterers in the turbid medium during a time interval τ. S(ρ) is the point source located at ρ; ρ is the source–detector distance. $\mu_{a}$ and $\mu_{s}^{\prime }$ are the tissue’s absorption and reduced scattering coefficients, respectively. For a semi-infinite medium, the solution of Eq. (1) using the extrapolated boundary condition for continuous-wave DCS is

$$G_{1}(\rho ,\tau )=\frac{3\mu_{s}^{\prime }}{4\pi }(\frac{\exp(-Kr_{1})}{r_{1}}-\frac{\exp(-Kr_{2})}{r_{2}}),$$ (2)

where $K=\sqrt{3\mu_{a}\mu_{s}^{\prime }+\alpha \mu_{s}^{\prime 2}k_{0}^{2}\langle \Delta r^{2}(\tau )\rangle }$, $r_{1}=\sqrt{\rho^{2}+z_{0}^{2}}$, $r_{2}=\sqrt{\rho^{2}+{\left(z_{0}+2z_{b}\right)}^{2}}$, $z_{0}={\left(\mu_{a}+\mu_{s}^{\prime }\right)}^{-1}$, and $z_{b}=5/(3\mu_{s}^{\prime })$ to be consistent with Ref. 46. Previous studies have shown that the scatters’ Brownian diffusion motion model^18^^,^^47^ aligns well with *in vivo* DCS experiments, and therefore, the mean-squared displacement can be derived as $\langle \Delta r^{2}(\tau )\rangle =6D_{b}\tau$, where $D_{b}$ represents the effective diffusion coefficient. BFi in DCS is typically defined as $\alpha D_{b}$.^21^^,^^48^ $g_{2}(\tau )$ is linked to the normalized electric field auto-correlation function as

$$g_{2}(\rho ,\tau )=1+\beta g_{1}{\left(\rho ,\tau \right)}^{2};\;g_{1}(\rho ,\tau )=|\frac{G_{1}(\rho ,\tau )}{G_{1}(\rho ,\tau =0)}|,$$ (3)

where β is a constant accounting for the collection setup, such as the number of detected speckles and the numerical aperture of the detection fiber.

**Fig. 2:**
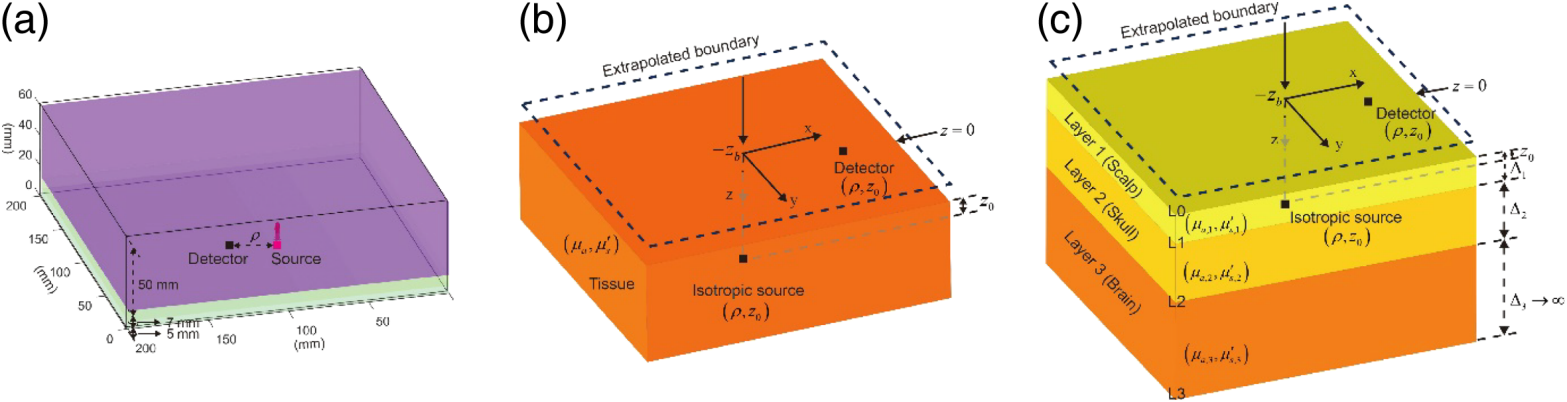
Simulation layered model and analytical models. (a) A large slab representing a human brain consisting of three layers of the scalp (5 mm), skull (7 mm), and brain (50 mm). (b) The homogenous semi-infinite analytical model used for fitting methods and generating deep-learning training datasets. (c) Three-layer geometric scheme including the position of the source and detector, each layer has its own thickness $\Delta_{1,2,3}$ and characterized by the absorption coefficient $\mu_{a1,2,3}$ and reduced scattering coefficient $\mu_{s1,2,3}^{\prime }$.

However, realistic biological tissues^49^ show multiple layers with different physiological and optical properties. Using DCS to conduct *in vivo* CBF measurements, light must propagate through different layers, including the scalp and skull.^50^^,^^51^ Thus, layered analytical models have been proposed for BFi extraction. These include the two-^27^^,^^28^ and three-layer analytical models.^24^^,^^30^^,^^31^^,^^52^ This study considers the three-layer analytical model, where a turbid medium consisting of N slabs is considered, as shown in Fig. 2(c). Each slab has its thickness, $\Delta_{p}=L_{p}-L_{p-1}$, p=1, 2, 3, where $L_{0,1,2,3}$ are the coordinates along the z-axis and $\mu_{a1,2,3}$, and $\mu_{s1,2,3}^{\prime }$ are absorption and scattering coefficients. To solve Eq. (1) in the layered medium (along z direction), we can use the Fourier transform G(r,τ) for the transverse coordinate ρ as

G^(q,z,τ)=∫d2ρG(r,τ)exp(iq·ρ), (4)

where q is the radial spatial frequency. Equation (1) can then be rewritten as

[∂2∂z2−Θ(p)2(q,τ)]G^(q,z,τ)=−3μs(p)′δ(z−z′), (5)

where $\Theta_{\left(p\right)}^{2}(q,\tau )=3\mu_{a(p)}\mu_{s(p)}^{\prime }+6k_{0}^{2}\mu_{s(p)}^{\prime 2}D_{b(p)}\tau +q^{2}$, $z^{\prime }=1/\mu_{s1}^{\prime }$, and p=1,2,3.

We divided the top layer into two sublayers: layer 0 ($0<z<z^{\prime }$) identified by p=0, and layer 1 ($z^{\prime }<z<\Delta_{1}$). Then, the solution of Eq. (5) inside the p’th layer (p=1,2,3) can be written as

G^(q,z,τ)=A(p) exp(Θ(p)z)+B(p) exp(−Θ(p)z), (6)

where $A_{\left(p\right)}$ and $B_{\left(p\right)}$ are coefficients for each layer determined by the boundary conditions

G^0(q,z,τ)−z0∂∂zG^0(q,z,τ)=0,z=0,G^0(q,z,τ)=G^1(q,z,τ),z=z′,∂∂zG^0(q,z,τ)=∂∂zG^1(q,z,τ)+3μs1′,z=z′,G^p(q,z,τ)=G^p+1(q,z,τ),z=Lp,  p=1,2,Dp∂∂zG^p(q,z,τ)=Dp+1∂∂zG^p+1(q,z,τ)z=Lp,  p=1,2,G^3(q,z,τ)+z3∂∂zG^3(q,z,τ)=0,z=L3, (7)

where $z_{0}∼1/\mu_{s1}^{\prime }$ and $z_{3}∼1/\mu_{s3}^{\prime }$ are the extrapolation lengths accounting for internal reflections at the tissue surface (z=0) and the back surface ($z=L_{3}$), respectively. $D_{p}=c/3\mu_{s(p)}^{\prime }$ is the photon diffusion coefficient in layer p, and c is the speed of light.

Substituting Eq. (6) into Eq. (7), $A_{\left(p\right)}$ and $B_{\left(p\right)}$ can be determined (p=1, 2, 3), and we obtain the solution of Eq. (5) at z=0 as

G^(q,z=0,τ)=NumDenom, (8)

where Num and Denom (when p=3 and $\Delta_{3}\to \infty$) are

$$\mathrm{Num}=3\mu_{s1}^{\prime }(z_{0}\Theta_{1}D_{1}\;\cosh(\Theta_{1}(\Delta_{1}-z^{\prime }))(\Theta_{2}D_{2}\;\cosh(\Theta_{2}\Delta_{2})+\Theta_{3}D_{3}\;\sinh(\Theta_{2}\Delta_{2}))+\Theta_{2}D_{2}(\Theta_{3}D_{3}\;\cosh(\Theta_{2}\Delta_{2})+\Theta_{2}D_{2}\;\sinh(\Theta_{2}\Delta_{2}))\sinh(\Theta_{1}(\Delta_{1}-z^{\prime }))),$$ (9)

$$\text{Denom}=\Theta_{2}D_{2}\;\cosh(\Theta_{2}D_{2})(\Theta_{1}(D_{1}+\Theta_{3}D_{3}z_{0})\;\cosh(\Theta_{1}D_{1})+(\Theta_{3}D_{3}+\;\Theta_{1}^{2}D_{1}z_{0})\sinh(\Theta_{1}D_{1}))+(\Theta_{1}(\Theta_{3}D_{1}D_{3}+\Theta_{2}^{2}D_{2}^{2}z_{0})\cosh(\Theta_{1}D_{1})+(\Theta_{2}^{2}D_{2}^{2}+\Theta_{1}^{2}\Theta_{3}D_{1}D_{3}z_{0})\;\sinh(\Theta_{1}D_{1})\;)\sinh(\Theta_{2}\Delta_{2}).$$ (10)

Therefore, by performing the inverse Fourier transform of Eq. (8) with respect to q, the field ACF at z=0 can be written as

G(ρ,z=0,τ)=1(2π)2∫d2qG^(q,z=0,τ)exp(−iq·ρ)=12π∫dqG^(q,z=0,τ)qJ0(q·ρ), (11)

$$g_{2}(\rho ,z=0,\tau =0)=\frac{G(\rho ,z=0,\tau )}{G(\rho ,z=0,\tau =0)},$$ (12)

where $J_{0}$ denotes the zero-order Bessel function of the first kind. The integral bound for q in Eq. (11) should theoretically be from 0 to +∞. However, in practice, the numerical integration is performed with a limited range as $\left[0\;{\mathrm{mm}}^{-1},30\;{\mathrm{mm}}^{-1}\right]$ advised in Ref. 29.

### 2.2 Noise Models

This study evaluates the impact from noise on BFi and β. We employed a broadly accepted noise model proposed by Zhou et al.^53^ The standard deviation (σ(τ), noise) of $g_{2}(\tau )$ is given as

$$\sigma (\tau )=\sqrt{\frac{T_{b}}{T_{\mathrm{int}}}}[\beta^{2}\frac{(1+e^{-2\Gamma T_{b}})(1+e^{-2\Gamma \tau })+2m(1-e^{-2\Gamma T_{b}})e^{-2\Gamma \tau }}{1-e^{-2\Gamma T_{b}}}\;+2{\left\langle n\right\rangle }^{-1}\beta (1+e^{-2\Gamma \tau })+{\left\langle n\right\rangle }^{-2}(1+e^{-\Gamma \tau }){]}^{1/2},$$ (13)

where $T_{b}$ is the bin width of the correlator, m is the bin index corresponding to τ. $\langle n\rangle \equiv IT_{b}$ is the average number of photons detected within the bin time, where I is the detected photon count rate, and $T_{\mathrm{int}}$ is the integration time (e.g., measurement duration). Γ is the decay rate of $g_{2}(\tau )$, which is obtained from fitting the measured $g_{2}(\tau )$ to the theoretical $g_{2}(\tau )\approx 1+\beta \;\exp(-\Gamma \tau )$. Gaussian noise^54^^,^^55^ was added to $g_{2}(\tau )$ based on a statistical noise model to determine the noise (σ(τ)). Considering realistic photon budgets, the photon count rate at 785 nm was assumed to be 8.05 kcps.^55^ Three different noise levels were defined according to $T_{\mathrm{int}}$ (= 1, 10, or 30 s).

### 2.3 Intrinsic Sensitivity Estimation

To evaluate the sensitivity to changes in blood flow in the deeper layer, we fixed the effective diffusion coefficient $D_{b}=1\times 10^{-6}\;{\mathrm{mm}}^{2}/s$ in layer 1 and increased $D_{b}$ in layer 3 as $\alpha D_{b}=[1+0.1\times (w-1)]\times 6\times 10^{-6}\;{\mathrm{mm}}^{2}/s$, w is an integer and w=1,2,…,11. The physiological and optical parameters listed in Table 1 are taken as baseline conditions. Similar to Ref. 54, the intrinsic sensitivity ($\eta_{H}$) is defined as

$$\eta_{H}=\frac{({\mathrm{BFi}}_{H}-{\mathrm{BFi}}_{0})/{\mathrm{BFi}}_{0}}{({\mathrm{CBF}}_{perturb}-{\mathrm{CBF}}_{0})/{\mathrm{CBF}}_{0}}\times 100\%,$$ (14)

where ${\mathrm{BFi}}_{H}$ and ${\mathrm{BFi}}_{0}$ represent the estimated BFi (H=D, S, or T, meaning DCS-NET, the semi-infinite, and three-layer fitting methods) for the perturbed and baseline conditions, respectively, and ${\mathrm{CBF}}_{perturb}$ and ${\mathrm{CBF}}_{0}$ are $D_{b}$ in layer 3 for the perturbed and baseline conditions, respectively.

**Table 1:** Physiological and optical parameters^56^ at 785 nm in the human head model.

Layer	Thickness (mm)	$\mu_{a}$ (${\mathrm{mm}}^{-1}$)	$\mu_{s}^{\prime }$ (${\mathrm{mm}}^{-1}$)	Blood flow index (${\mathrm{mm}}^{2}/s$)
Scalp ($\Delta_{1}$)	5	0.019	0.660	$1\times 10^{-6}$
Skull ($\Delta_{2}$)	7	0.014	0.860	0
Brain	50	0.019	1.110	$6\times 10^{-6}$

### 2.4 Monte Carlo Simulations

We utilized a simplified model comprising three layers to emulate the scalp (5 mm), skull (7 mm), and brain (50 mm, large enough so that we can treat the medium as semi-infinite), respectively.^57^ All layers were assumed homogeneous, as demonstrated in Fig. 2(a), and their corresponding optical properties are summarized in Table 1.

MCX utilized an anisotropic factor (g) of 0.89 and a refractive index (n) of 1.37^44^ for all layers. We launched $2\times 10^{9}$ photons from a source with a diameter of 1 mm and set the detector radii to 0.13, 0.28, 0.45, 0.7, 1, and 1.5 mm for ρ=5, 10, 15, 20, 25, and 30 mm, respectively, recording data from multiple distances simultaneously. An example of the source and the detector was arranged as shown in Fig. 2(a). MCX records the path lengths and momentum transfer from the detected photons for obtaining the electric field ACF $G_{1}(\tau )$:^22^

$$G_{1}(\tau )=\frac{1}{N_{p}}\overset{N_{p}}{\underset{s=1}{\sum }}\;\exp(-\frac{1}{3}k_{0}^{2}\overset{N_{t}}{\underset{i=1}{\sum }}Y_{s,i}{\left\langle \Delta r^{2}(\tau )\right\rangle }_{i})\exp(-\overset{N_{t}}{\underset{i=1}{\sum }}\mu_{a,i}L_{s,i}),$$ (15)

where $N_{p}$ is the number of detected photons, $N_{t}$ is the number of tissue types (3 for our simulations), and $Y_{s,i}$ and $L_{s,i}$ stand for the total momentum transfer and the total path length of photon s in layer i, respectively. $\mu_{a,i}$ is the absorption coefficient, and ${\left\langle \Delta r^{2}(\tau )\right\rangle }_{i}$ is the mean square displacement of the scattered particles in layer i. Here, ${\left\langle \Delta r^{2}(\tau )\right\rangle }_{i}=6D_{i}\tau$, where $D_{i}$ is the effective diffusion coefficient of layer i. The simulated $G_{1}(\tau )$ is normalized to $G_{1}(0)$, and then we can obtain $g_{2}(\tau )$ using the Siegert relationship with β=0.5. In this simulation, the delay time 1  μs≤τ<10,000  μs (127 data points) was used for $g_{2}(\tau )$.

### 2.5 Deep Learning Architecture Design

**Fig. 3:**
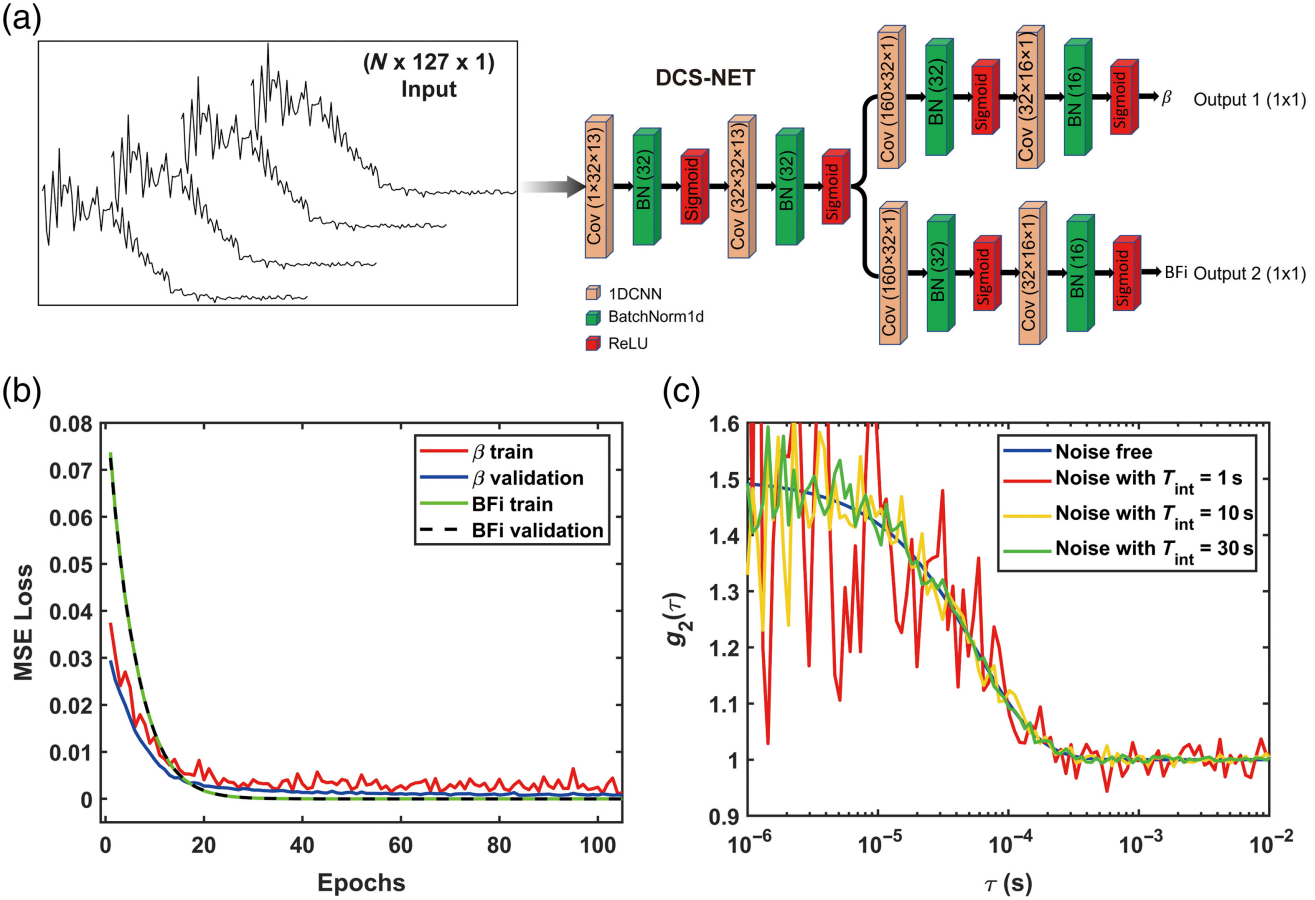
Design and evaluation of the convolution neural network (CNN). (a) The proposed DCS-NET includes a CNN, BN, and sigmoid activation layers. The convolution layer parameters are the filter number × the kernel size × the stride. (b) Training and validation losses of DCS-NET. (c) $g_{2}(\tau )$ with noise-free (blue), and with realistic noise added, assuming an 8.05 kcps at 785 nm at different noise levels with $T_{\mathrm{int}}=1$, 10, and 30 s.

The structure of DCS-NET is shown in Fig. 3(a). DCS-NET takes $g_{2}(\tau )$ to estimate β and BFi independently. DCS-NET consists of (1) a shared branch for temporal feature extraction and (2) two subsequent independent branches for estimating β and BFi, with a similar structure to the shared branch. The two CNN layers in the shared branch have a wider sliding window with a larger kernel size of 13 and a giant stride of 5. They are expected to capture more general features of the auto-correlation decay curves. The batch normalization (BN) layer^58^ is employed after each convolutional layer. It reduces the shift of internal covariance and accelerates network training when processing normalized data. To implement feature pooling and effectively reconstruct β and BFi, we use a pointwise convolution layer with a kernel size of 1 after the convolutional neural network, followed by the activation function, the Sigmoid function. The model input is measured (here, we used data from MCX) $g_{2}(\tau )$, of which the size is 1×127. Both the estimated β and BFi have a size of 1×1. Note that the simulated $g_{2}(\tau )$ was normalized to (0, 1] before being fed into the model.

### 2.6 Training Dataset Preparation

The training datasets can be easily obtained using synthetic data based on the homogenous semi-infinite analytical model, as shown in Fig. 2(b). Thus, according to Eqs. (2) and (3), 200,000 training datasets (200,000×127) were generated and split into the training (80%) and the validation (20%) groups. Each dataset consists of the input, $g_{2}(\tau )$, and its corresponding labels are BFi and β, which are the output. The training batch size is 128, with 800 training epochs. We used an early stopping callback with 20 patient epochs to prevent overfitting. To match the realistic experiments, in the dataset, we set $\mu_{a}\in U(0.01,1]\;{\mathrm{mm}}^{-1}$, $\mu_{s}^{\prime }\in U(0.5,1.6]\;{\mathrm{mm}}^{-1}$, β∈U(0,1], $\mathrm{BFi}\in [10^{-8},10^{-5}]{\;\mathrm{mm}}^{2}/s$, and ρ∈U[5,30]  mm, where U stands for a uniform distribution. $g_{2}(\tau )$ training datasets contain noisy and noiseless (the noise model has been described in Ref. 53) ACFs, as shown in Fig. 3(c). The green, yellow, and red lines represent noisy $g_{2}(\tau )$, and the blue line represents noiseless $g_{2}(\tau )$. We used the optimizer Adam^59^ for the training process, with the learning rate fixed at $1\times 10^{-5}$ in the standard back-propagation. We used the mean square error loss function for updating the network by controlling the following problem:

$$L(𝒫)=\frac{1}{M}\overset{M}{\underset{i}{\sum }}{\left\|F(X^{i},𝒫)-Y^{i}\right\|}_{2}^{2},$$ (16)

where X is the network output (estimated BFi or β), and Y is the corresponding label (true BFi or β) in the i’th training pairs. F is the mapping function, 𝒫 is the trainable weights of our networks, and M is the number of training pairs. Figure 3(b) shows that the training and validation losses decrease rapidly and reach the plateau after 85 epochs. The training process’s best score reaches a small value of 0.000725, indicating that the network is well trained as the estimated β and BFi are close to the ground truth. The model was conducted in Python using Pytorch with Intel (R) Core (TM) i9-10900KF CPU @3.70 GHz.

## 3 Results

### 3.1 Absolute BFi Recovery Versus Detection Depths

To investigate how the absolute BFi and β behave in terms of ρ among DCS-NET, semi-infinite, and three-layer fitting approaches, we generated $g_{2}(\tau )$ via MCX Monte Carlo simulations for ρ=5, 10, 15, 20, 25, and 30 mm, as described in Sec. 2.4. Table 1 shows all the relevant parameters used in MCX simulations. The absolute BFi in this study corresponds to the Brownian diffusion coefficient $D_{b}$ (assumed α=1). When using DCS-NET, $g_{2}(\tau )$ was fed into the pre-trained model. For the semi-infinite fitting procedure, $g_{2}(\tau )$ was fitted to Eqs. (2) and (3), and we assumed $\mu_{a}=0.019\;{\mathrm{mm}}^{-1}$, $\mu_{s}^{\prime }=1.099\;{\mathrm{mm}}^{-1}$, for the brain layer (layer 3), as provided in Table 1.

We also fitted the simulated $g_{2}(\tau )$ with the three-layer model, Eqs. (11) and (12), and $D_{b1}=1\times 10^{-6}\;{\mathrm{mm}}^{2}/s$, $D_{b2}=0{\;\mathrm{mm}}^{2}/s$, $D_{b3}=6\times 10^{-6}{\;\mathrm{mm}}^{2}/s$, $\mu_{a1}=0.019{\;\mathrm{mm}}^{-1}$, $\mu_{s1}^{\prime }=0.635{\;\mathrm{mm}}^{-1}$, $\mu_{a2}=0.014{\;\mathrm{mm}}^{-1}$, $\mu_{s2}^{\prime }=0.851\;\mathrm{mm}/s$, $\mu_{a3}=0.019{\;\mathrm{mm}}^{-1}$, $\mu_{s3}^{\prime }=1.099{\;\mathrm{mm}}^{-1}$, $\Delta_{1}=5\;\mathrm{mm}$, and $\Delta_{2}=7\;\mathrm{mm}$. Meanwhile, we set β=0.3 and $D_{b3}=2\times 10^{-7}\;{\mathrm{mm}}^{2}/s$ as the initial guesses. For the fitting, we used NLSM (lsqcurvefit(·) in MATLAB with the Levenberg–Marquardt optimization) to minimize the unweighted least squares objective function,

$$\arg\;\min\;\overset{j=N_{\tau }}{\underset{j=1}{\sum }}{\left[g_{2}(\tau {)}_{\mathrm{MCX}}-g_{2}(\tau {)}_{H}\right]}^{2},\;H=(S,T),$$ (17)

where $N_{\tau }$ is the number of sampled $g_{2}(\tau )$, and $g_{2}{\left(\tau \right)}_{H}$ is from Eq. (3) or Eq. (12). Fitting was performed on τ from 1 to 10,000  μs.

Table 2 presents the true β and BFi and estimated β and BFi using DCS-NET, semi-infinite, and three-layer fitting methods. All input parameters for fitting are assumed as described above, and $\beta_{\mathrm{GT}}=0.5$. We define ${\mathrm{BFi}}_{D}$, ${\mathrm{BFi}}_{S}$, and ${\mathrm{BFi}}_{T}$ (also $\beta_{D}$, $\beta_{S}$, and $\beta_{T}$) for DCS-NET, the semi-infinite, and three-layer fitting methods, respectively. We define $\varepsilon_{\mathrm{BFi},D}(\%)=|{\mathrm{BFi}}_{D}-{\mathrm{BFi}}_{\mathrm{GT}}|/{\mathrm{BFi}}_{\mathrm{GT}}\times 100\%$, where $\varepsilon_{\mathrm{BFi},D}$ is the BFi error with DCS-NET. Similarly, $\varepsilon_{\mathrm{BFi},S}$ and $\varepsilon_{\mathrm{BFi},T}$ are the BFi estimated errors with the semi-infinite and three-layer fitting methods.

**Table 2:** BFi in the brain estimated using DCS-NET, homogeneous semi-infinite and three- layer fitting models.

ρ (mm)	Layer	${\mathrm{BFi}}_{\mathrm{GT}}$ (${\mathrm{mm}}^{2}/s$)	${\mathrm{BFi}}_{D}$ (${\mathrm{mm}}^{2}/s$)	BFi estimated by fitting methods (${\mathrm{mm}}^{2}/s$)	
				${\mathrm{BFi}}_{S}$	${\mathrm{BFi}}_{T}$
5	1	$1\times 10^{-6}$	$\beta_{D}=0.521$	$\beta_{S}=0.501$	$\beta_{T}=0.493$
	2	0	${\mathrm{BFi}}_{D}=8.45\times 10^{-7}$	${\mathrm{BFi}}_{S}=7.15\times 10^{-7}$	${\mathrm{BFi}}_{T}=7.15\times 10^{-7}$
	3	$6\times 10^{-6}$			
10	1	$1\times 10^{-6}$	$\beta_{D}=0.509$	$\beta_{S}=0.499$	$\beta_{T}=0.493$
	2	0	${\mathrm{BFi}}_{D}=7.36\times 10^{-7}$	${\mathrm{BFi}}_{S}=5.47\times 10^{-7}$	${\mathrm{BFi}}_{T}=2.17\times 10^{-5}$
	3	$6\times 10^{-6}$			
15	1	$1\times 10^{-6}$	$\beta_{D}=0.501$	$\beta_{S}=0.498$	$\beta_{T}=0.504$
	2	0	${\mathrm{BFi}}_{D}=1.03\times 10^{-6}$	${\mathrm{BFi}}_{S}=4.79\times 10^{-7}$	${\mathrm{BFi}}_{T}=1.43\times 10^{-5}$
	3	$6\times 10^{-6}$			
20	1	$1\times 10^{-6}$	$\beta_{D}=0.499$	$\beta_{S}=0.495$	$\beta_{T}=0.506$
	2	0	${\mathrm{BFi}}_{D}=2.07\times 10^{-6}$	${\mathrm{BFi}}_{S}=4.57\times 10^{-7}$	${\mathrm{BFi}}_{T}=8.17\times 10^{-6}$
	3	$6\times 10^{-6}$			
25	1	$1\times 10^{-6}$	$\beta_{D}=0.499$	$\beta_{S}=0.493$	$\beta_{T}=0.505$
	2	0	${\mathrm{BFi}}_{D}=4.82\times 10^{-6}$	${\mathrm{BFi}}_{S}=4.63\times 10^{-7}$	${\mathrm{BFi}}_{T}=5.63\times 10^{-6}$
	3	$6\times 10^{-6}$			
30	1	$1\times 10^{-6}$	$\beta_{D}=0.499$	$\beta_{S}=0.491$	$\beta_{T}=0.505$
	2	0	${\mathrm{BFi}}_{D}=5.71\times 10^{-6}$	${\mathrm{BFi}}_{S}=4.88\times 10^{-7}$	${\mathrm{BFi}}_{T}=4.97\times 10^{-6}$
	3	$6\times 10^{-6}$			

Table 2 shows when the semi-infinite model is used, the estimated BFi is closer to layer 1 ($\alpha D_{b}=1\times 10^{-6}\;{\mathrm{mm}}^{2}/s$), even for ρ=30  mm, suggesting that a homogenous fitting procedure is more sensitive to the superficial layers’ dynamic properties. This finding is consistent with the results reported by Gagnon *et al*.^27^ Using the three-layer fitting model, we obtained ${\mathrm{BFi}}_{T}=7.15\times 10^{-7}\;{\mathrm{mm}}^{2}/s$, close to $1\times 10^{-6}\;{\mathrm{mm}}^{2}/s$ when ρ=5  mm. This is because the mean light penetration depth is ∼ρ/3 to ρ/2.^19^ When ρ is small, most detected photons predominantly travel through layer 1. As ρ increases (ρ≥10  mm), the estimated BFi decreases, reaching $5.63\times 10^{-6}\;{\mathrm{mm}}^{2}/s$ at ρ=25  mm, with $\varepsilon_{\mathrm{BFi},T}$ of 6.17%. This is because as ρ increases, the detected photons penetrate inside the skull layer ($\alpha D_{b}=0\;{\mathrm{mm}}^{2}/s$), resulting in an increased contribution of layer 2. This phenomenon is expected, because the three-layer modeling can remove the contribution from superficial layers^52^ to obtain accurate BFi. Interestingly, when using DCS-NET, the estimated BFi increases as ρ increases, reaching $5.71\times 10^{-6}\;{\mathrm{mm}}^{2}/s$ with $\varepsilon_{\mathrm{BFi},D}$ of 4.83% at ρ=30  mm. These results suggest that the AI model is capable of recognizing the depth. Regarding β estimation, there is no significant difference among the three methods.

### 3.2 Absolute BFi Recovery with Noise

**Fig. 4:**
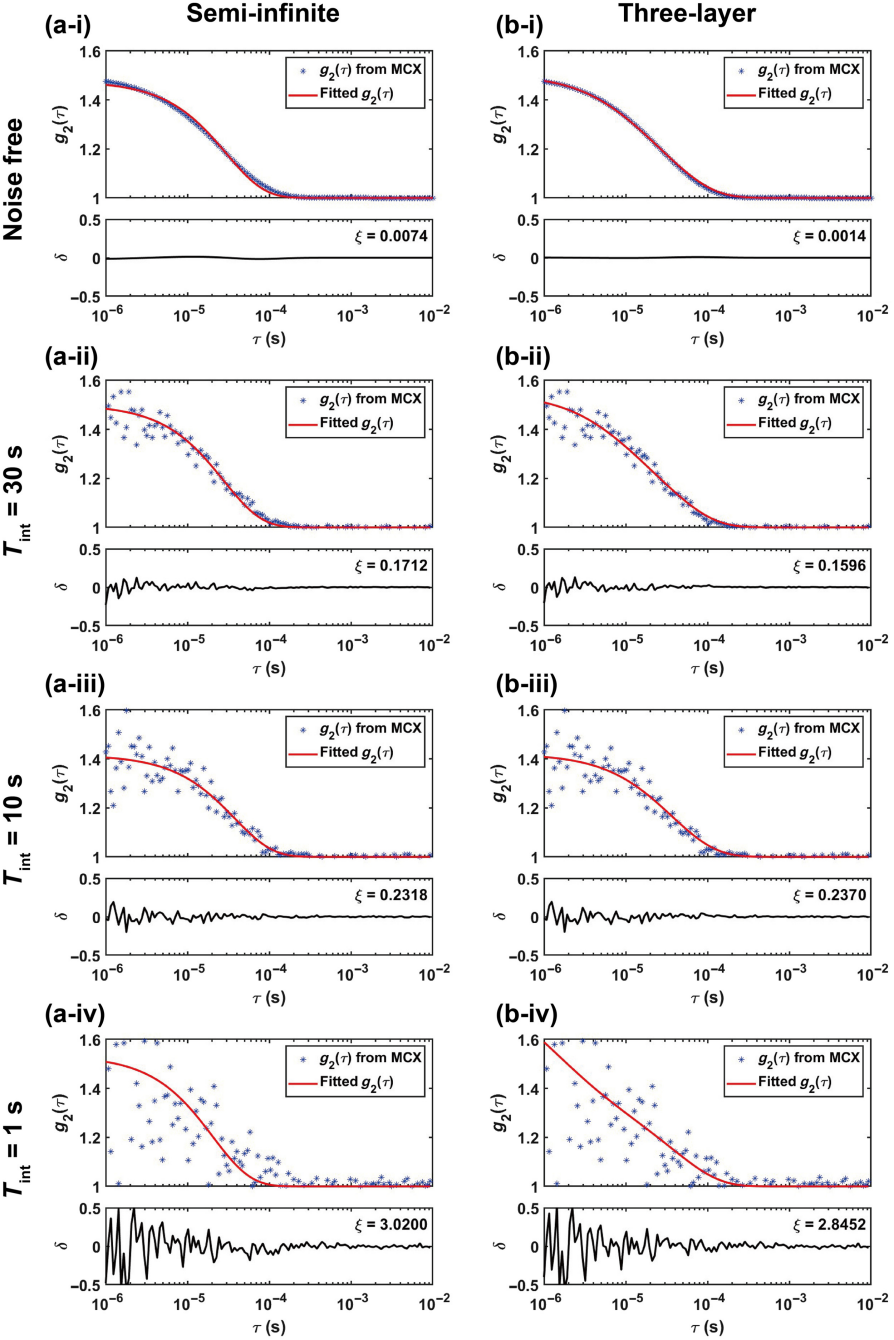
MCX-generated (scattered stars) and fitted (red solid lines) $g_{2}$ curves using semi-infinite and three-layer fitting methods. [(a) (i)–(iv), respectively] noisy MCX simulated data (scattered star-shaped) at different noise levels fitted with the semi-infinite homogeneous model; [(b) (i)–(iv)] noisy MCX-generated data fitted with the for the three-layer fitting procedure. The corresponding residual δ and resnorm ϵ curves are also included.

Figure 3(c) displays the semi-infinite analytical example $g_{2}(\tau )$ curves with noise using the model proposed by Zhou et al.^53^ The curves were obtained with ρ=30  mm at different noise levels ($T_{\mathrm{int}}=1,10,30\;s$), $\mu_{a}=0.019\;{\mathrm{mm}}^{-1}$, and $\mu_{s}^{\prime }=1.099\;{\mathrm{mm}}^{-1}$ with an assumed $\mathrm{BFi}=2\times 10^{-7}\;{\mathrm{mm}}^{2}/s$. To assess DCS-NET’s performance in practical scenarios, we modified the Monte Carlo code to generate $g_{2}$ curves including noise according to Zhou et al.’s noise model.^53^ We generated 100 $g_{2}$ sets for each noise level (including noiseless). Still, we minimized Eq. (17) using the Levenberg–Marquardt optimization routine. We performed the residual analysis to assess the efficiency of the semi-infinite and three-layer models. We define the residual δ and resnorm (the squared 2-norm of the residual) ϵ as

$$\delta =f(\beta ,\mathrm{BFi},\tau_{q})-g_{2}(\tau_{q}),\;\epsilon =\overset{q=Q}{\underset{q=1}{\sum }}\delta^{2},$$ (18)

where q is the lag time index, and Q is the length of the time trace. $f(\beta ,\mathrm{BFi},\tau_{q})$ is the fitted $g_{2}(\tau )$ obtained from fitting methods based on analytical models at the lag time $\tau_{q}$, and the corresponding true value is $g_{2}(\tau_{q})$ from MCX. The fitting results using the semi-infinite and three-layer analytical models are presented in Fig. 4, in which noisy $g_{2}(\tau )$ curves from MCX (blue star-shaped) and fitted $g_{2}(\tau )$ curves (red lines) at different noise levels are shown. Figures 4(a)(i)–4(a)(iv) show the MCX-generated and fitted $g_{2}$ using the semi-infinite model, and they exhibit an increasing trend in δ, ranging from (−0.0025,0.0025) to (−0.5,0.5), indicating that the semi-infinite method becomes inaccurate when the noise level increases. Additionally, ϵ reaches 3.02 when $T_{\mathrm{int}}=1\;s$. Similar behaviors are observed in the three-layer fitting, as shown in Figs. 4(b)(i)–4(b)(iv).

**Fig. 5:**
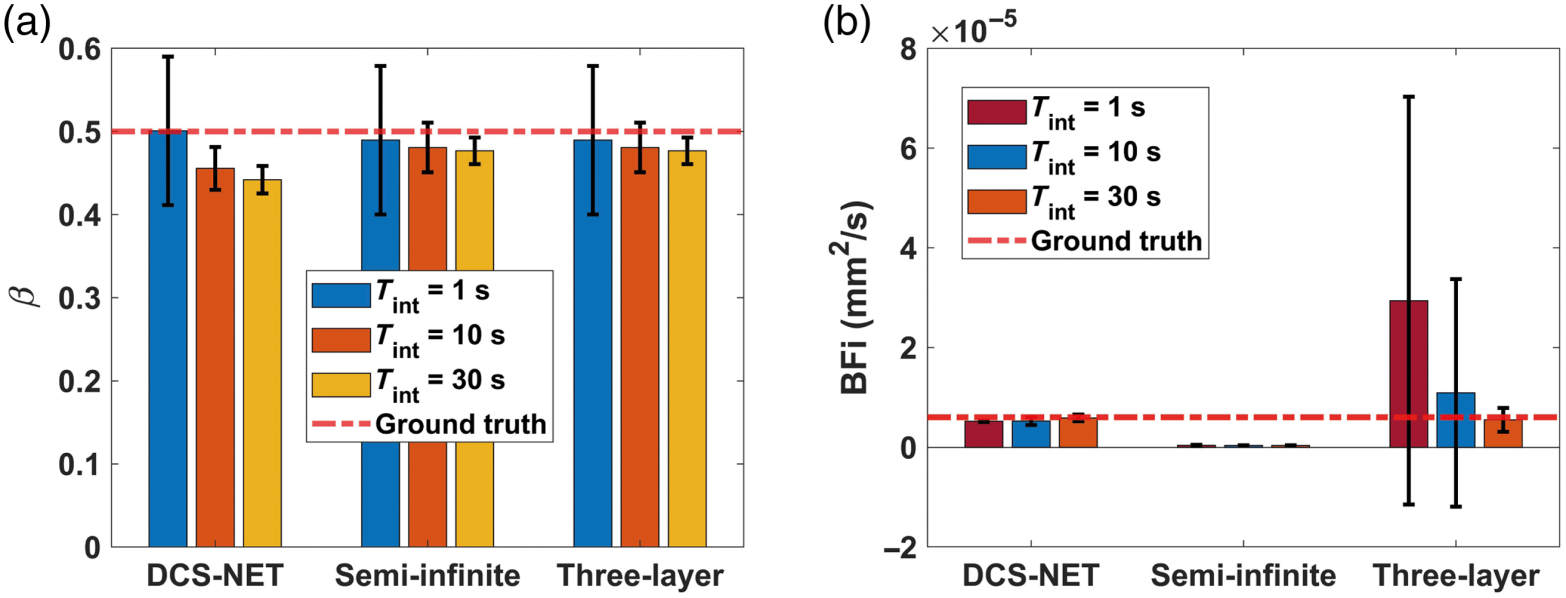
Estimated β by DCS-NET, semi-infinite, and three-layer fitting methods at different noise levels ($T_{\mathrm{int}}=1$, 10, and 30 s). The bar height means the average value for estimated BFi or β, the error bar means the standard deviations σ. (b) The estimated BFi by the three methods at different noise levels. The red dot line stands for the ground truth. (All the average values were obtained over 100 trials.)

We also calculated the mean BFi and β over 100 trials. As for β, we arrive at the same conclusion as Sec. 3.1 that all three methods exhibit similar behaviors at the same noise level. A high noise level ($T_{\mathrm{int}}=1\;s$) leads to a significant standard deviation, as shown in Fig. 5(a). Figure 5(b) shows the estimated BFi. The estimated BFi for the semi-infinite model deviates significantly from the ground truth. When using the three-layer fitting method, $\varepsilon_{\mathrm{BFi},T}$ is 82.30% at the lower noise level ($T_{\mathrm{int}}=30\;s$). As the noise level increases, $\varepsilon_{\mathrm{BFi},T}$ also increases, with $\varepsilon_{\mathrm{BFi},T}$ reaching 390.10% at the high noise level ($T_{\mathrm{int}}=1\;s$). Furthermore, a high noise level leads to a more significant standard deviation, indicating that BFi estimation is highly sensitive to noise when the three-layer fitting method is applied, in accordance with previous findings.^52^ In contrast, $\varepsilon_{\mathrm{BFi},D}$ (using DCS-NET) at a high noise level ($T_{\mathrm{int}}=1\;s$) is 12.87%, whereas at a low noise level ($T_{\mathrm{int}}=30\;s$), it is only 1.93%, indicating that DCS-NET is not susceptible to noise. Figure 5(b) also shows that when the three-layer fitting method is used, the BFi precision can be enhanced through increasing $T_{\mathrm{int}}$.

### 3.3 Relative Blood Flow

**Fig. 6:**
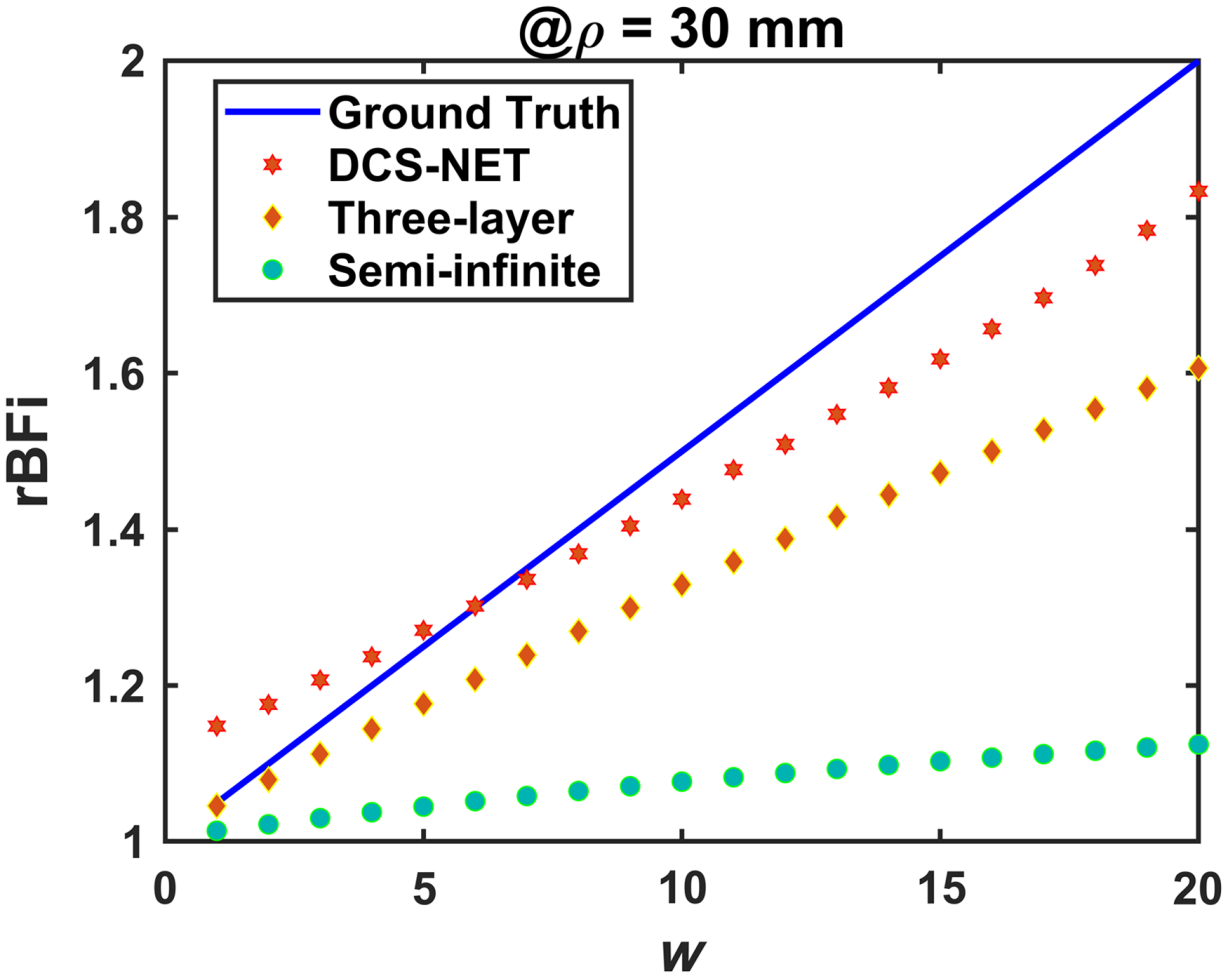
rBFi calculated by DCS-NET, the semi-infinite, and three-layer fitting methods on noiseless data for ρ=30  mm for $\alpha D_{b}$ ranges from $6\times 10^{-6}\;{\mathrm{mm}}^{2}/s$ to $1.2\times 10^{-5}\;{\mathrm{mm}}^{2}/s$ (w=1,…,21) with a step of $0.05\times 10^{-6}$. $\mathrm{rBFi}=\mathrm{BFi}/{\mathrm{BFi}}_{0}$, we define the estimated BFi as ${\mathrm{BFi}}_{0}$ at the start point.

In practice, we do not aim to obtain absolute BFi measurements. Instead, the relative variation in blood flow (e.g., $\mathrm{rBFi}=\mathrm{BFi}/{\mathrm{BFi}}_{0}$) is oftener used.^19^ To evaluate DCS-NET for extracting rBFi in the brain, we assigned $\alpha D_{b}(w)=[1+0.05\times (w-1)]\times 6\times 10^{-6}\;{\mathrm{mm}}^{2}/s$, w=1,2,…,21 in layer 3 (brain) and fixed $\alpha D_{b}$ in other layers. Figure 6 presents rBFi calculated on noiseless data at ρ=30  mm.

In Fig. 6, rBFi calculated by DCS-NET, the semi-infinite, and three-layer fitting methods on noiseless data for ρ=30  mm for $\alpha D_{b}$ ranges from $6\times 10^{-6}$ to $1.2\times 10^{-5}\;{\mathrm{mm}}^{2}/s$ (w=1,…,21) with a step of $0.05\times 10^{-6}$. $\mathrm{rBFi}=\mathrm{BFi}/{\mathrm{BFi}}_{0}$, we define the estimated BFi as ${\mathrm{BFi}}_{0}$ at the start point.

To compare the accuracy of the three different methods in quantifying rBFi, we defined the error in rBFi as $\varepsilon_{\mathrm{rBFi},H}=|{\mathrm{rBFi}}_{H}-{\mathrm{rBFi}}_{\mathrm{GT}}|/{\mathrm{rBFi}}_{\mathrm{GT}}\times 100\%$ (H=D, S, or T), meaning the rBFi estimation error using DCS-NET, the semi-infinite, and three-layer fitting methods, respectively. We can observe that ${\mathrm{rBFi}}_{D}$ (red star) is close to the true rBFi (blue solid line) with $\varepsilon_{\mathrm{rBFi},D}$ ranging from 0.15% to 8.35%. By contrast, the semi-infinite and three-layer methods result in more significant errors of $3.41\%\leq \varepsilon_{\mathrm{rBFi},S}\leq 43.76\%$ and $0.36\%\leq \varepsilon_{\mathrm{rBFi},T}\leq 19.66\%$, respectively. As expected, the semi-infinite homogenous solution resulted in significant errors in rBFi, in agreement with Ref. 33.

### 3.4 Intrinsic Sensitivity

**Fig. 7:**
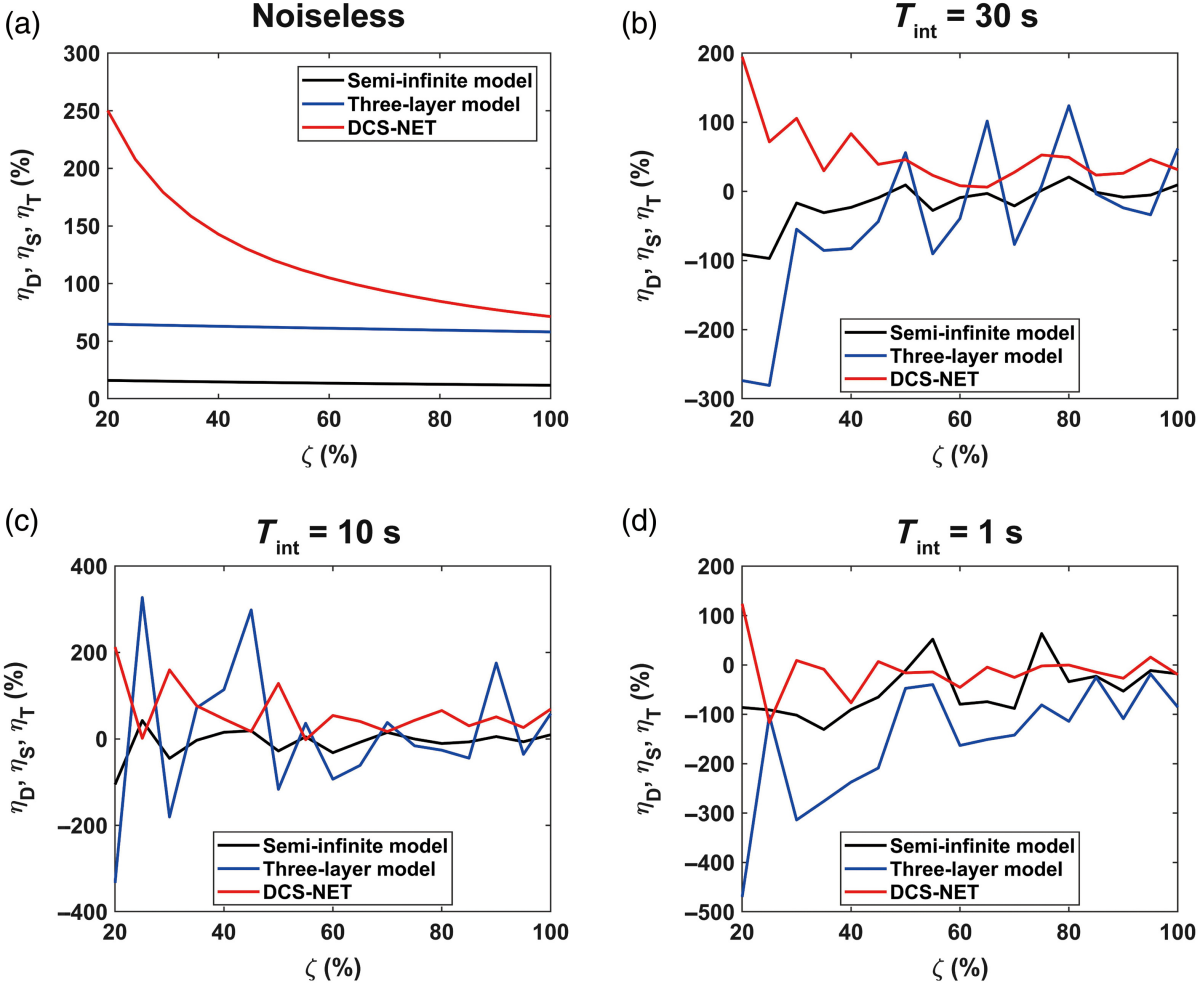
(a) Intrinsic sensitivity on noiseless data. (b)–(d) The sensitivities for noise with $T_{\mathrm{int}}=30\;s$, $T_{\mathrm{int}}=10\;s$, $T_{\mathrm{int}}=1\;s$, respectively. η is the intrinsic sensitivity that defined in Eq. (14), and ζ is the perturbation level in layer 3 (brain). Red, blue, and dark lines present $\eta_{D}$, $\eta_{T}$, and $\eta_{S}$, respectively. The perturbation levels in the graphs start at ζ=20%.

As described in Sec. 2.3, the input $D_{b}$ in layer 3, denoted as ${\mathrm{CBF}}_{0}=6\times 10^{-6}\;{\mathrm{mm}}^{2}/s$, serves as the base point, and its corresponding recovered BFi is denoted as ${\mathrm{BFi}}_{0}$. Similarly, we assigned $\alpha D_{b}=[1+0.05\times (w-1)]\times 6\times 10^{-6}\;{\mathrm{mm}}^{2}/s$ (w is an integer; w=1,2,…,21), and it is referred to as the perturbed blood flow ${\mathrm{CBF}}_{perturb}$. We also define a perturbation level $\zeta =({\mathrm{CBF}}_{perturb}-{\mathrm{CBF}}_{0})/{\mathrm{CBF}}_{0}\times 100\%$. We calculated the corresponding BFi for $\alpha D_{b}$, and then used Eq. (14) to obtain $\eta_{D}$, $\eta_{S}$ and $\eta_{T}$. We considered physiological noise by utilizing the noise model described in Sec. 2.2. Figure 7(a) shows the noiseless intrinsic sensitivity, demonstrating that DCS-NET exhibits $\eta_{D}>71.34\%$. The intrinsic sensitivity reaches 2.5 × when ζ=20%, then decreases with ζ increasing. In comparison, the three-layer fitting method achieved $\eta_{T}=61.96\%$, whereas the semi-infinite fitting method yielded $\eta_{S}$ of only 14.12% on noiseless data. Figures 7(b)–7(d) illustrate sensitivity curves at various noise levels. Especially noteworthy are the instances where $\eta_{D}\;>0$ at $T_{\mathrm{int}}=10\;s$ and $T_{\mathrm{int}}=30\;s$. Conversely, with the semi-infinite and three-layer fitting models, η predominantly assumes negative values, underscoring the considerable impact of measurement noise on sensitivity. Furthermore, the impact of measurement noise on the sensitivity overgrows, particularly for the three-layer fitting method, as apparent in Fig. 7(d).

### 3.5 BFi Extraction with Varied Optical Properties and Scalp/Skull Thicknesses

In practical applications, a patient’s head parameters can vary significantly, and the ideal scenario is to measure them before conducting DCS measurements. However, it is not always straightforward, and we usually assume average values. However, we must evaluate the impact of assumed errors on BFi estimation. Since $\mu_{a}$ and $\mu_{s}^{\prime }$ are typically unknown and have to be measured separately or taken from literature. We examined how $\mu_{a}$ and $\mu_{s}^{\prime }$ of layer 3 (brain) impact BFi extraction. Changing the scalp/skull thickness also varies BFi, which can be observed using the multi-layered model fitting method. Here, we use the three-layer fitting method, and all BFi were obtained at ρ=30  mm. Additional details are presented in Table 3.

**Table 3:** Varying optical properties and scalp ($\Delta_{1}$) and skull ($\Delta_{2}$) thicknesses.

	−40%	−20%	0%	+20%	+40%
$\mu_{a}$ (${\mathrm{mm}}^{-1}$)	0.011	0.015	0.019	0.023	0.027
$\mu_{s}^{\prime }$ (${\mathrm{mm}}^{-1}$)	0.659	0.879	1.099	1.319	1.539
$\Delta_{1}$ (mm)	3.000	4.000	5.000	6.000	7.000
$\Delta_{2}$ (mm)	4.200	5.600	7.000	8.400	9.800

#### 3.5.1 $\mu_{a}$ variation

To study how $\mu_{a}$ impacts BFi, we set $\mu_{a}=0.011$, 0.015, 0.019, 0.023, and 0.027 and $\mu_{s}^{\prime }=1.099{\;\mathrm{mm}}^{-1}$ in MCX. The baseline is at $\mu_{a}=0.019\;{\mathrm{mm}}^{-1}$, with ±20% and ±40% variation. In this case, two BFi groups were calculated. The first group was calculated assuming a constant $\mu_{a}=0.019{\;\mathrm{mm}}^{-1}$ (0%), defined as $\mu_{a,m}$, and the calculated BFi is defined as ${\mathrm{BFi}}_{m}$. The second group was calculated using the known $\mu_{a}$ set in MCX, which we considered as true $\mu_{a}$, and the corresponding calculated BFi is considered as ${\mathrm{BFi}}_{\mathrm{GT}}$.

#### 3.5.2 $\mu_{s}^{\prime }$ variation

Similarly, we conducted simulations with $\mu_{s}^{\prime }=0.666$, 0.888, 1.110, 1.332, and $1.554\;{\mathrm{mm}}^{-1}$ and a fixed $\mu_{a}=0.019{\;\mathrm{mm}}^{-1}$ to investigate how $\mu_{s}^{\prime }$ impacts BFi estimation. We define the estimated BFi as ${\mathrm{BFi}}_{m}$ when $\mu_{s}^{\prime }=1.099{\;\mathrm{mm}}^{-1}$ (at 0%, defined as $\mu_{s,m}^{\prime }$). Additionally, ${\mathrm{BFi}}_{\mathrm{GT}}$ was calculated using the known $\mu_{s}^{\prime }$ set in MCX, considered as true $\mu_{s}^{\prime }$.

**Fig. 8:**
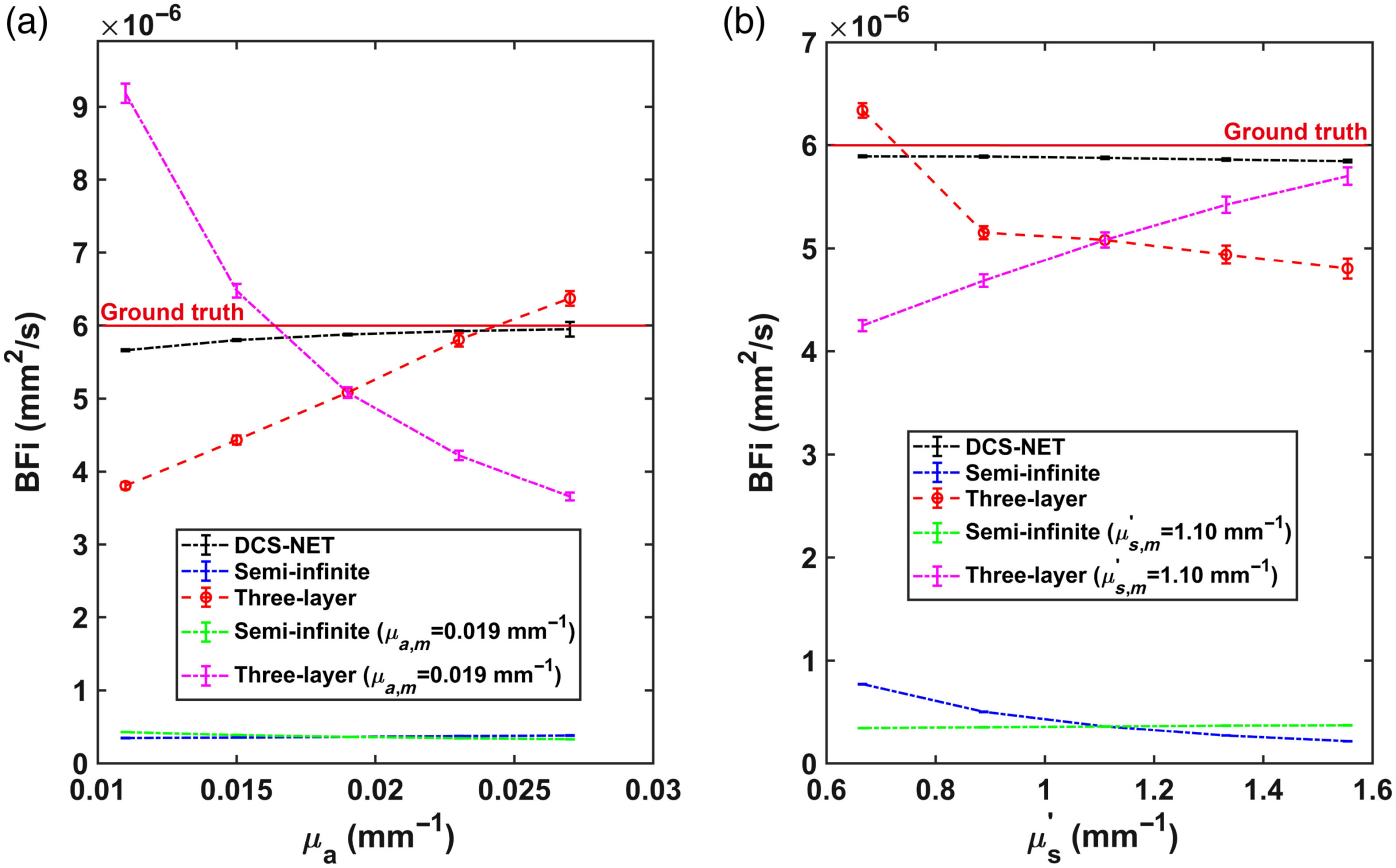
(a) Estimated BFi versus $\mu_{a}$, the green and purple dashed lines are for ${\mathrm{BFi}}_{m}$ assuming $\mu_{a}=0.019{\;\mathrm{mm}}^{-1}$, the red solid and black dashed lines are for ${\mathrm{BFi}}_{\mathrm{GT}}$ and ${\mathrm{BFi}}_{D}$, respectively, and the red and blue dashed lines are for ${\mathrm{BFi}}_{\mathrm{GT}}$ using the three-layer and semi-infinite fitting methods. (b) Estimated BFi versus $\mu_{s}^{\prime }$, the green and purple dashed lines are for ${\mathrm{BFi}}_{m}$ assuming $\mu_{s}^{\prime }=1.10{\;\mathrm{mm}}^{-1}$, the red solid and black dashed lines are for ${\mathrm{BFi}}_{\mathrm{GT}}$ and ${\mathrm{BFi}}_{D}$, respectively, and the red and blue dashed lines are for ${\mathrm{BFi}}_{\mathrm{GT}}$ using the three-layer and semi-infinite fitting methods.

The mean and standard deviation of the estimated BFi (versus $\mu_{a}$) over 100 trials are shown in Fig. 8(a). We also compare ${\mathrm{BFi}}_{m}$ and ${\mathrm{BFi}}_{\mathrm{GT}}$. The blue (${\mathrm{BFi}}_{\mathrm{GT}}$) and green (${\mathrm{BFi}}_{m}$) dashed lines are for the semi-infinite model, whereas the red (${\mathrm{BFi}}_{\mathrm{GT}}$) and purple (${\mathrm{BFi}}_{m}$) dashed line are for the three-layer model. The red solid (${\mathrm{BFi}}_{\mathrm{GT}}$) and black dashed lines are for DCS-NET. Similarly, the BFi’s mean and standard deviation (versus $\mu_{s}^{\prime }$) over 100 trials are shown in Fig. 8(b).

**Fig. 9:**
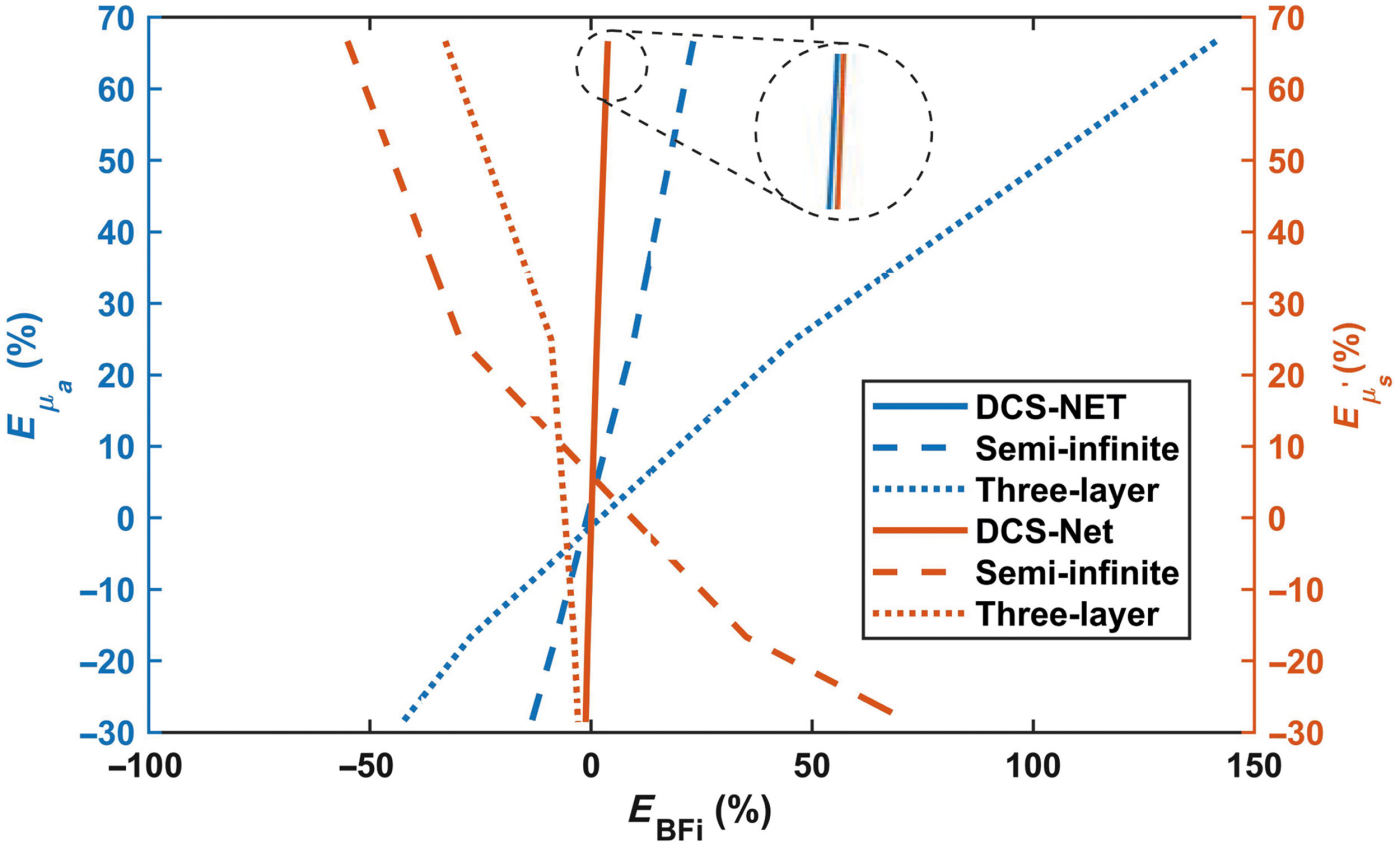
BFi error (in %) versus errors in the $\mu_{a}$ and $\mu_{s}^{\prime }$ variation (in %) among DCS-NET, semi-infinite, and three-layer fitting methods.

Figure 9 shows the BFi variation (in %) versus the $\mu_{a}$ and $\mu_{s}^{\prime }$ variations (in %). The percentage error for $\mu_{a}$ is defined as $E_{\mu_{a}}=[\frac{\mu_{a,m}-\mu_{a}}{\mu_{a}}]\times 100\%$. Similarly, we define the percentage error for $\mu_{s}^{\prime }$ as $E_{\mu_{s}^{\prime }}=[\frac{\mu_{s,m}^{\prime }-\mu_{s}^{\prime }}{\mu_{s}^{\prime }}]\times 100\%$. The BFi error (in %) caused by assumed error in $E_{\mu_{a}}$ or $E_{\mu_{s}^{\prime }}$ is defined as $E_{\mathrm{BFi}}=[\frac{{\mathrm{BFi}}_{m}-{\mathrm{BFi}}_{\mathrm{GT}}}{{\mathrm{BFi}}_{\mathrm{GT}}}]\times 100\%$.

Figures 8 and 9 show that $E_{\mathrm{BFi}}$ is positively related to $E_{\mu_{a}}$ and negatively related to $E_{\mu_{s}^{\prime }}$ for semi-infinite and three-layer fitting models, in good agreement with previous findings.^26^^,^^31^ On the other hand, $E_{\mathrm{BFi}}$ curves obtained from DCS-NET are close and are not sensitive to $E_{\mu_{a}}$ and $E_{\mu_{s}^{\prime }}$. This result is expected, as from Eq. (2), $\mu_{s}^{\prime }$ should yield a more pronounced impact compared to $\mu_{a}$, primarily due to the second-order contribution from $\mu_{s}^{\prime }$ and $\mu_{s}^{\prime }\gg \mu_{a}$ observed in biological tissues. Extreme $E_{\mathrm{BFi}}$ examples are shown in Fig. 9, namely, a more extensive $E_{\mu_{a}}∼+62\%$ results in $E_{\mathrm{BFi}}∼+25\%$ and $E_{\mu_{a}}∼-30\%$ results in $E_{\mathrm{BFi}}∼-10\%$. When $E_{\mu_{s}^{\prime }}$ reaches +62%, $E_{\mathrm{BFi}}$ reaches ∼−50% and $E_{\mu_{s}^{\prime }}∼-30\%$ gives $E_{\mathrm{BFi}}∼+70\%$.

The results from the three-layer fitting model show similar behaviors. Namely, $E_{\mathrm{BFi}}$ is positively related to $E_{\mu_{a}}$   and negatively related to $E_{\mu_{s}^{\prime }}$ in layer 3, this result aligns well with the conclusions from Zhao et al.’ conclusion.^31^ In contrast, DCS-NET only shows −1%∼+5% in $E_{\mathrm{BFi}}$ caused by $E_{\mu_{a}}$ and $E_{\mu_{s}^{\prime }}$ (blue solid and brown solid lines for $\mu_{a}$ and $\mu_{s}^{\prime }$, respectively in Fig. 9), indicating that the variations in $\mu_{a}$ and $\mu_{s}^{\prime }$ have negligible impact on BFi estimation.

#### 3.5.3 Scalp thickness variation

To investigate $\Delta_{1}$’s impact on BFi, we varied $\Delta_{1}$ (= 3, 4, 5, 6, and 7 mm) and fixed $\Delta_{2}=7\;\mathrm{mm}$ in MCX. We define the estimated BFi as ${\mathrm{BFi}}_{m}$ when $\Delta_{1}=5\;\mathrm{mm}$ (0%, defined as $\Delta_{1,m}$). Additionally, ${\mathrm{BFi}}_{\mathrm{GT}}$ was calculated using the known $\Delta_{1}$ set in MCX, considered as true $\Delta_{1}$.

#### 3.5.4 Skull thickness variation

Similarly, to investigate $\Delta_{2}$’s impact on BFi, we varied $\Delta_{2}$ (= 4.2, 5.6, 7.0, 8.4, and 9.8 mm) and fixed $\Delta_{1}=5\;\mathrm{mm}$ in MCX. We define the estimated BFi as ${\mathrm{BFi}}_{m}$ calculated when $\Delta_{2}=7.0\;\mathrm{mm}$ (0%, defined as $\Delta_{2,m}$). Additionally, ${\mathrm{BFi}}_{\mathrm{GT}}$ was calculated using the known $\Delta_{2}$ set in MCX, considered as true $\Delta_{2}$.

**Fig. 10:**
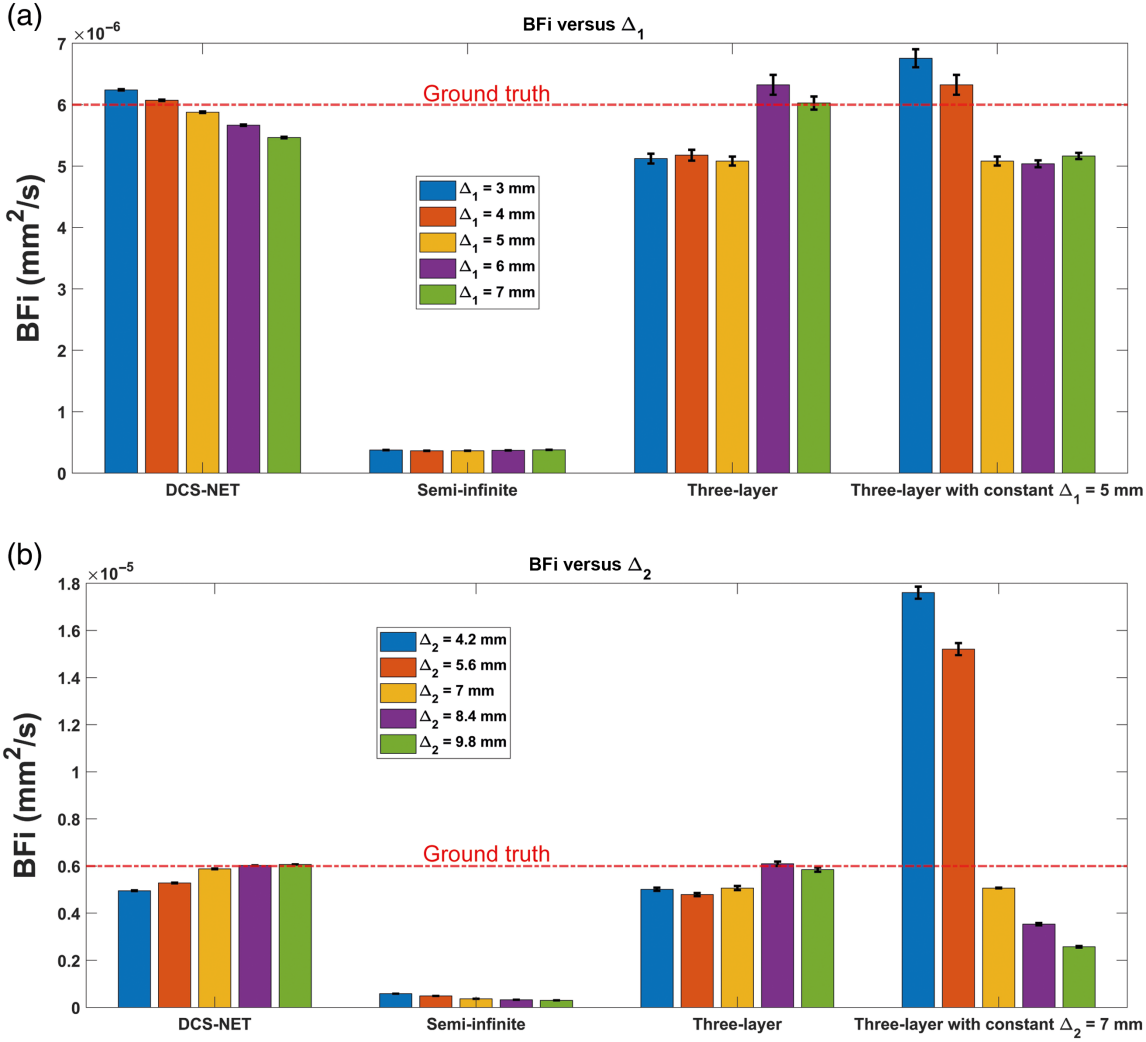
(a) BFi’s mean value and standard deviation versus $\Delta_{1}$, and the rightmost bar group represents the results obtained with $\Delta_{1}=5\;\mathrm{mm}$. (b) BFi’s mean value and standard deviation versus $\Delta_{2}$, and the rightmost bar group represents the results obtained with $\Delta_{2}=7\;\mathrm{mm}$. Each bar in the plot represents the average BFi over 100 trials calculated using three different methods, whereas the error bar stands for the standard deviation of BFi over 100 trials.

Figure 10(a) presents BFi’s mean value (represented by bar plots) and standard deviation (depicted by error bars) over 100 trials versus $\Delta_{1}$. The rightmost bar group represents the results obtained with $\Delta_{1}=5\;\mathrm{mm}$. Figure 10(b) shows BFi’s mean value and standard deviation versus $\Delta_{2}$, the rightmost bar group represents the results obtained with $\Delta_{2}=7\;\mathrm{mm}$. Still, we can see that the semi-infinite model cannot provide accurate BFi at a deeper layer. When $\Delta_{1}$ changed, $\varepsilon_{\mathrm{BFi},D}$ falls into 1.17%∼8.33% [the bar group 1 in Fig. 10(a)] when using DCS-NET, whereas $\varepsilon_{\mathrm{BFi},T}$ falls into 4.30%∼14.66% [the bar group 3 in Fig. 10(a)] using the three-layer fitting model, slightly larger than that using DCS-NET. However, $\varepsilon_{\mathrm{BFi},T}$ increases to 11.67%∼16.05% when $\Delta_{1}$ estimation error occurs using the three-layer fitting method [shown in the rightmost bar group in Fig. 10(a)]. Whereas for the variation in $\Delta_{2}$, $\varepsilon_{\mathrm{BFi},D}$ falls into 0.33%∼10.33% when DCS-NET is used [the bar group 1 in Fig. 10(b)], whereas $\varepsilon_{\mathrm{BFi},T}$ falls into 1.50%∼13.33% when the three-layer fitting method is used [the bar group 3 in Fig. 10(b)]. Both present similar accuracy. However, when $\Delta_{2}$ is not accurate, $\varepsilon_{\mathrm{BFi},T}$ becomes more pronounced and reaches 41.09%∼193.40% [the rightmost bar group in Fig. 10(b)].

**Fig. 11:**
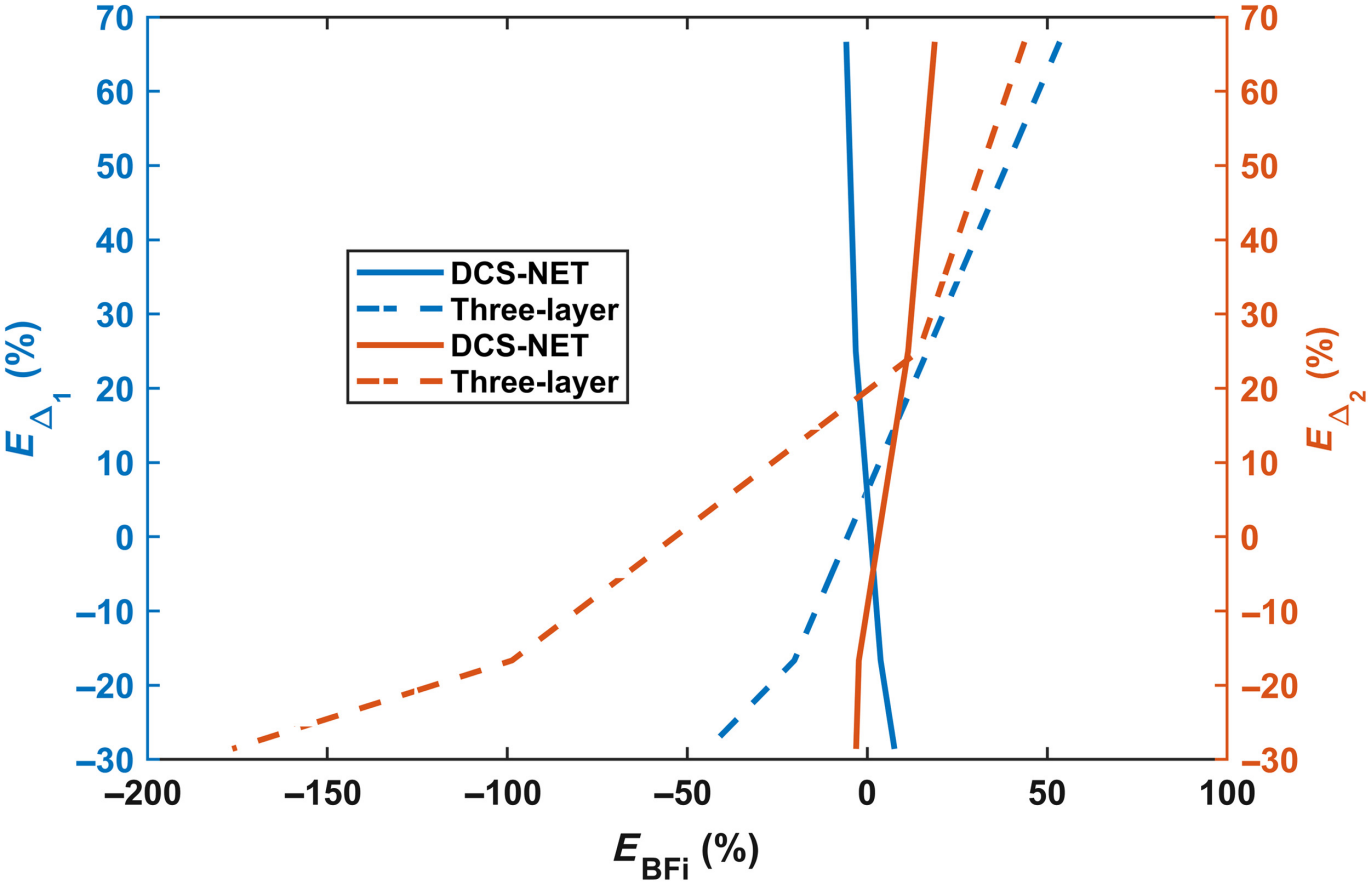
BFi error (in %) versus errors in $\Delta_{1}$ and $\Delta_{2}$ (in %) between DCS-NET and three-layer fitting methods.

Figure 11 shows the BFi variation (in %) versus the $\Delta_{1}$ and $\Delta_{2}$ variations (in %). The percentage error for $\Delta_{1}$ is defined as $E_{\Delta_{1}}=[\frac{\Delta_{1,m}-\Delta_{1}}{\Delta_{1}}]\times 100\%$. Similarly, we define the percentage error for $\Delta_{2}$ as $E_{\Delta_{2}}=[\frac{\Delta_{2,m}-\Delta_{2}}{\Delta_{2}}]\times 100\%$. The BFi error (in %) caused by assumed error in $E_{\Delta_{1}}$ and $E_{\Delta_{2}}$ is defined as $E_{\mathrm{BFi}}=[\frac{{\mathrm{BFi}}_{m}-{\mathrm{BFi}}_{\mathrm{GT}}}{{\mathrm{BFi}}_{\mathrm{GT}}}]\times 100\%$.

As it is commonly known, $E_{\Delta_{1}}$ and $E_{\Delta_{2}}$ cause a significant $E_{\mathrm{BFi}}$. Figures 10(a) and 10(b) demonstrate a positive correlation between $E_{\mathrm{BFi}}$ and $E_{\Delta_{1}}$ (and $E_{\Delta_{2}}$). Furthermore, as observed in Fig. 11, $E_{\mathrm{BFi}}$ resulting from $E_{\Delta_{2}}$ ranges from −176.41% to +43.68%. In contrast, $E_{\mathrm{BFi}}$ caused by $E_{\Delta_{1}}$ ranges from −44.29% to +53.47%. This error range is significantly narrower than that caused by the skull thickness, agreeing with the findings in Ref. 31. For DCS-NET, $E_{\mathrm{BFi}}$ caused by both $\Delta_{1}$ and $\Delta_{2}$ falls within the limited range of −6% to +8%.

### 3.6 BFi Estimation Time

In addition, the BFi estimation time is also an important parameter, especially in real-time measurements, and Table 4 compares the three extraction methods. We record it for single decays and batch decays (e.g., 100 trials) at different noise levels. It is clear that DCS-NET is promising for real-time applications. All data reported in Table 4 are standard deviations and means for repeating three times after discarding the first few runs that usually take longer. The analysis were performed using the workstation (CPU: Intel(R) Core(TM) i9-10900X @3.70 GHz; Memory: 128 GB; graphics processing unit (GPU): NVIDIA Quadro RTX 5000).

**Table 4:** The BFi estimation time (with Matlab parfor for semi-infinite and three-layer fitting models).

Noise level	1 trial			100 trials		
	DCS-NET (s)	Semi-infinite (s)	Three-layer (s)	DCS-NET (s)	Semi-infinite (s)	Three-layer (s)
$T_{\mathrm{int}}=30\;s$	$0.001\pm 4.567\times 10^{-4}$	0.034±0.012	17.141±2.027	$0.004\pm 8.728\times 10^{-4}$	0.159±0.030	180.176±3.029
$T_{\mathrm{int}}=10\;s$	$0.001\pm 2.846\times 10^{-4}$	0.031±0.021	16.946±3.157	$0.004\pm 7.367\times 10^{-4}$	0.157±0.029	181.023±2.025
$T_{\mathrm{int}}=1\;s$	$0.001\pm 2.544\times 10^{-4}$	0.030±0.039	17.946±4.587	$0.004\pm 2.765\times 10^{-4}$	0.160±0.032	187.118±5.398
Noiseless	$0.001\pm 3.007\times 10^{-4}$	0.032±0.001	17.002±1.248	$0.004\pm 9.415\times 10^{-4}$	0.156±0.012	180.169±3.017

## 4 Discussion

Our study shows that DCS-NET can robustly quantify DCS-based blood flow measurements. We used DCS-NET to analyze the ACFs generated from MCX. The proposed network is based on 1DCNN,^43^ which is straightforward, quicker to train, and faster than high-dimension CNNs for time sequence analysis, such as FLIM data.^43^^,^^60^ To evaluate DCS-NET, we compared it with the semi-infinite, three-layer fitting methods by changing tissue optical properties ($\mu_{a}$ and $\mu_{s}^{\prime }$), depths (related to ρ), and scalp/skull thicknesses ($\Delta_{1}$ and $\Delta_{2}$). BFi estimated by DCS-NET shows a small error range −1%∼+5% induced by $\mu_{a}$ and $\mu_{s}^{\prime }$ (see Fig. 9) and a slightly wider error range −6%∼+8% induced by $\Delta_{1}$ and $\Delta_{2}$ (see Fig. 11). For rBFi, the error from DCS-NET (8.35%) is much less than that of the semi-infinite and three-layer fitting methods (43.76% and 19.66%, respectively). Moreover, DCS-NET yields more than 71.34% sensitivity to brain blood flow, whereas the semi-infinite and three-layer fitting methods yield 14.12% and 61.96%, respectively [Fig. 7(a)]. We considered measurement noise using a stochastic noise model^53^ to reflect experimental realities. With DCS-NET, $\varepsilon_{\mathrm{BFi},D}$ is 12.87% at a high noise level ($T_{\mathrm{int}}=1\;s$), whereas it increases to 390.10% when using the three-layer fitting method. At a low noise level ($T_{\mathrm{int}}=30\;s$), the three-layer fitting model yields $\varepsilon_{\mathrm{BFi},T}$ of 82.30%, much worse than 1.93% obtained by DCS-NET, suggesting that DCS-NET is less sensitive to noise [see Fig. 5(b)]. Figures 10(a) and 10(b) show that the three-layer analytical method (modeling the head, i.e., scalp, skull, and brain) can minimize the influence of extracerebral layers on measured DCS signals. However, this model requires a priori knowledge of layer optical properties and thicknesses. Therefore, accurately estimating scalp and skull thicknesses is required for reliable CBF estimation when using a three-layer analytical model.

Besides accuracy and robustness, the computational cost is a critical factor that impacts practical applications, especially for real-time monitoring. Table 4 reveals that it took 0.004 s for DCS-NET to quantify 100 $g_{2}$ curves with 127 data points. In contrast, it took 0.160 and 181.697 s, respectively, for the semi-infinite fitting and three-layer fitting procedures. For quantifying a single autocorrelation decay curve, it only took 0.001 s for DCS-NET. In contrast, it took 0.032 and 17.496 s, respectively, for the semi-infinite fitting and three-layer fitting procedures. DCS-NET is the fastest among the three, around 17,000-fold faster than the three-layer model and 32-fold faster than the semi-infinite model.

Table 5 lists existing deep learning methods applied to DCS techniques. It shows that DCS-NET’s training is much faster than 2DCNNs,^40^ approximately 140-fold faster. Although the remaining models, RNN,^39^ LSTM,^41^ and ConvGRU,^42^ have fewer total layers, they are limited to a specific ρ.

**Table 5:** Comparison of existing AI methods for BFi estimation.

Model	Training parameters	Training time	Total layer	ρ (mm)	Year
DCS-NET	25,506	∼13 min	18	5 to 30	2023
RNN^39^	174,080	N/A	20	25	2019
CNN(2D)^40^	75,552	∼30.5 h	161	27.5	2020
LSTM^41^	1161	N/A	2	15	2021
ConvGRU^42^	11,557	N/A	10	20	2022

Notes: the training parameters of RNN and CNN(2D) are not given in the literature; we calculate them according to the structure shown in the literature.

Although DCS-NET is more robust than the semi-infinite and three-layer fitting methods, our study has several limitations. First, DCS-NET’s training datasets were generated using the semi-infinite diffusion model as advised in Ref. 40. Nevertheless, this model does not consider scalp and skull thicknesses, which could potentially explain why the error range (−6%∼+8%) caused by $\Delta_{1}$ and $\Delta_{2}$ is much broader than that (−1%∼+5%) caused by $\mu_{a}$ and $\mu_{s}^{\prime }$ (Figs. 9 and 11). The complexity of including training datasets generated from a layered model is beyond the scope of this study, given this report’s already long length. In future, we will train new networks using datasets generated from a layered model, and alternatively, obtaining training datasets from *in vivo* measurements, as demonstrated in Refs. 41 and 42 will also be considered. Second, current rBFi calculations do not consider variations in optical properties between the baseline and activation states. Indeed, $\mu_{a}$ and $\mu_{s}^{\prime }$ in the brain can vary according to interventions (e.g., functional activation), which are recognized to impact perfusion. Failing to account for these changes could introduce additional uncertainties in rBFi measurements. Third, we did not include a comparison with the two-layered analytical model in this report; it may be worth further investigation. Fourth, as we all know, analytical fitting methods suffer from partial volume effects and recover only a fraction of the actual change; still, the relationship between the recovered change and the actual change remains linear. However, from Fig. 7(a), we can see the BFi values from DCS-NET reflect various degrees of the relative ground truth change according to the relative change; thus, they have a non-linear relationship with actual brain blood flow. This suggests processing data with our DCS-NET could result in non-physiological distortions. We will further investigate this and improve our network models in future studies. Finally, our study was solely conducted using simulation data. In the future, we will perform phantom and *in vivo* experiments to validate our findings.

## 5 Conclusion

We compared the proposed DCS-NET against the semi-infinite and the three-layer models for estimating β, BFi and rBFi. We used Monte Carlo simulations to validate their performances. This study evaluated the cerebral sensitivity using a deep learning method and the influence of scalp/skull thickness and $\mu_{a}/\mu_{s}^{\prime }$ variations on BFi extraction. Additionally, we examined the impact of noise. Our findings revealed that the homogenous model is sensitive to superficial layers. In contrast, the three-layer model performs better in estimating BFi in deeper layers but is more susceptible to measurement noise.

Furthermore, DCS-NET outperforms the semi-infinite and three-layer fitting models in rBFi recovery. Using DCS-NET, variations in $\mu_{a}$ and $\mu_{s}^{\prime }$ have less impact on BFi, unlike variations in scalp and skull thicknesses, which show a more significant error in BFi. Moreover, iterative fitting methods are much slower and unsuitable for real-time “online” processing. In contrast, our DCS-NET is 32-fold faster than the semi-infinite model and 17,000-fold faster than the three-layer model, showing great potential for continuous real-time clinical applications.

## 6 Appendix

Table 6 shows all essential parameters used in the throughout article, ensuring accessibility to comprehensive details for interested readers.

**Table 6:** Essential parameters list.

Variable	Variable full names	
α	The fraction of dynamic photon scattering events in the medium	
β	Coherent factor	
$\mu_{a}$	Absorption coefficient	
$\eta_{D}$	Intrinsic sensitivity using DCS-NET	Defined in Sec. 2.3
$\eta_{S}$	Intrinsic sensitivity using the semi-infinite fitting method	Defined in Sec. 2.3
$\eta_{T}$	Intrinsic sensitivity using the three-layer fitting method	Defined in Sec. 2.3
$E_{\mu_{a}}$	The percentage error of the assumed $\mu_{a}$	Defined in Sec. 3.5
$\mu_{s}^{\prime }$	Reduced scattering coefficient	
$E_{\mu_{s}^{\prime }}$	The percentage error of the assumed $\mu_{s}^{\prime }$	Defined in Sec. 3.5
ρ	Source–detector distance	
$D_{b}$	Brownian diffusion coefficient	
$D_{p}$	Photon diffusion coefficient	
q	Radial spatial frequency	
BFi	Blood flow index estimated in DCS (i.e., $\alpha D_{b}$)	
${\mathrm{BFi}}_{0}$	Baseline BFi	
${\mathrm{BFi}}_{m}$	Estimated BFi when assumed $\mu_{a}$, $\mu_{s}^{\prime }$, $\Delta_{1}$, $\Delta_{2}$ are constant at 0%	Defined in Sec. 3.5
${\mathrm{BFi}}_{\mathrm{GT}}$	Ground-truth blood flow	Defined in Sec. 3.5
${\mathrm{BFi}}_{D}$	Blood flow index estimated by DCS-NET	Defined in Sec. 3.1
${\mathrm{BFi}}_{S}$	Blood flow index estimated by the semi-infinite fitting method	Defined in Sec. 3.1
${\mathrm{BFi}}_{T}$	Blood flow index estimated by the three-layer fitting method	Defined in Sec. 3.1
$E_{\mathrm{BFi}}$ (%)	The BFi error (in %) between ${\mathrm{BFi}}_{m}$ and ${\mathrm{BFi}}_{\mathrm{GT}}$	Defined in Sec. 3.5
$\varepsilon_{\mathrm{BFi},D}$	Error percentage of BFi using DCS-NET	Defined in Sec. 3.1
$\varepsilon_{\mathrm{BFi},S}$	Error percentage of BFi using the semi-infinite fitting method	Defined in Sec. 3.1
$\varepsilon_{\mathrm{BFi},T}$	Error percentage of BFi using the three-layer fitting method	Defined in Sec. 3.1
rBFi	Relative blood flow index	Defined in Sec. 3.3
${\mathrm{rBFi}}_{\mathrm{GT}}$	Relative blood flow index for ground truth	Defined in Sec. 3.3
${\mathrm{rBFi}}_{D}$	rBFi estimated by DCS-NET	Defined in Sec. 3.3
$rBFi_{S}$	rBFi estimated by the semi-infinite fitting method	Defined in Sec. 3.3
${\mathrm{rBFi}}_{T}$	rBFi estimated by the three-layer fitting method	Defined in Sec. 3.3
$\varepsilon_{\mathrm{rBFi},D}$	Error percentage of rBFi using DCS-NET	Defined in Sec. 3.3
$\varepsilon_{\mathrm{rBFi},S}$	Error percentage of rBFi using the semi-infinite fitting method	Defined in Sec. 3.3
$\varepsilon_{\mathrm{rBFi},T}$	Error percentage of rBFi using the three-layer fitting method	Defined in Sec. 3.3
CBF	Cerebral blood flow	Defined in Sec. 3.4
${\mathrm{CBF}}_{0}$	Baseline cerebral blood flow	Defined in Sec. 3.4
${\mathrm{CBF}}_{perturb}$	$\alpha D_{b}$ during perturbed conditions	Defined in Sec. 3.4
ζ	Perturbation level	Defined in Sec. 3.4
$\Delta_{1}$	Scalp thickness	
$\Delta_{2}$	Skull thickness	
$T_{\mathrm{int}}$	Integration time	
$T_{b}$	The bin width of the correlator	
m	Bin index	
τ	Lag time	
$g_{1}$	Normalized electric auto-correlation function	
$g_{2}$	Normalized intensity auto-correlation function	
Γ	Decay rate of $g_{2}$	
w	Integer, w=1,2,3	
δ	Residual from fitting procedures	Defined in Eq. (18)
ϵ	Resnorm (the square 2-norm of the residual)	Defined in Eq. (18)

## Data Availability

The data and code supporting the findings of this study are available from the corresponding author upon reasonable request.
